# Variation in the vulnerability of mice expressing human superoxide dismutase 1 to prion-like seeding: a study of the influence of primary amino acid sequence

**DOI:** 10.1186/s40478-021-01191-w

**Published:** 2021-05-20

**Authors:** Jacob I. Ayers, Guilian Xu, Kristy Dillon, Qing Lu, Zhijuan Chen, John Beckman, Alma K. Moreno-Romero, Diana L. Zamora, Ahmad Galaleldeen, David R. Borchelt

**Affiliations:** 1 Department of Neuroscience, Center for Translational Research in Neurodegenerative Disease (CTRND), University of Florida, Box 100159, Gainesville, FL 32610 USA; 2 Institute for Neurodegenerative Disease, Weill Institute for Neurosciences, University of California, San Francisco, CA 94143 USA; 3 Department of Neurology, Weill Institute for Neurosciences, University of California, San Francisco, CA 94143 USA; 4 Department of Biological Sciences, St. Marys University, San Antonio, TX 78228 USA; 5 SantaFe HealthCare Alzheimers Disease Research Center, McKnight Brain Institute, University of Florida, Gainesville, FL 32610 USA

## Abstract

Misfolded forms of superoxide dismutase 1 (SOD1) with mutations associated with familial amyotrophic lateral sclerosis (fALS) exhibit prion characteristics, including the ability to act as seeds to accelerate motor neuron disease in mouse models. A key feature of infectious prion seeding is that the efficiency of transmission is governed by the primary sequence of prion protein (PrP). Isologous seeding, where the sequence of the PrP in the seed matches that of the host, is generally much more efficient than when there is a sequence mis-match. Here, we used paradigms in which mutant SOD1 seeding homogenates were injected intraspinally in newborn mice or into the sciatic nerve of adult mice, to assess the influence of SOD1 primary sequence on seeding efficiency. We observed a spectrum of seeding efficiencies depending upon both the SOD1 expressed by mice injected with seeds and the origin of the seed preparations. Mice expressing WT human SOD1 or the disease variant G37R were resistant to isologous seeding. Mice expressing G93A SOD1 were also largely resistant to isologous seeding, with limited success in one line of mice that express at low levels. By contrast, mice expressing human G85R-SOD1 were highly susceptible to isologous seeding but resistant to heterologous seeding by homogenates from paralyzed mice over-expressing mouse SOD1-G86R. In other seeding experiments with G85R SOD1:YFP mice, we observed that homogenates from paralyzed animals expressing the H46R or G37R variants of human SOD1 were less effective than seeds prepared from mice expressing the human G93A variant. These sequence mis-match effects were less pronounced when we used purified recombinant SOD1 that had been fibrilized in vitro as the seeding preparation. Collectively, our findings demonstrate diversity in the abilities of ALS variants of SOD1 to initiate or sustain prion-like propagation of misfolded conformations that produce motor neuron disease.

**Supplementary Information:**

The online version contains supplementary material available at 10.1186/s40478-021-01191-w.

## Introduction

Amyotrophic lateral sclerosis (ALS) can present clinically as focal weakness in a limb, hand, or foot that progressively worsens before weakness spreads along anatomically connected pathways, leading to generalized paralysis [1]. In a subset of cases, weakness first appears in muscles of the head and neck before spreading to include the diaphragm and intercostal muscles. The symptoms of weakness originate from dysfunction of both upper and lower motor neurons, with dysfunction of the upper neurons causing symptoms of spasticity before paralysis makes voluntary movements impossible. The vast majority of ALS cases have no clear etiology and no obvious family history; however, up to 25% of patients have a family history of disease with a subset of these classified as familial based on the inheritance of rare genetic variants [2]. Approximately 15% of familial ALS (fALS) and 2% of sporadic cases are associated with mutations in the gene encoding the antioxidant enzyme known as superoxide dismutase 1 (*SOD1*) [2]. To date, more than 170 missense mutations at more than 80 different amino acids of this 153 amino acid protein have been associated with ALS (https://alsod.uk). Though the effects of most of these mutations on enzymatic function have not been characterized, studies of a random subset of mutations have shown that many disease-associated mutants of SOD1 retain high enzymatic activity [3]. These mutants have been classified as wild-type-like (WT-like) mutants. Other mutations, however, can be highly destabilizing to the normal protein conformation and render the enzyme inactive [3]. Work from multiple laboratories including ours has demonstrated that mutations associated with ALS cause conformational changes in SOD1 that induce the protein to misfold and self-associate into insoluble aggregates and pathological inclusions (reviewed in [4]). Indeed, SOD1 inclusion pathology is a common feature of SOD1-linked ALS [5]. Whether misfolded SOD1 is also a common feature of sporadic ALS is less certain as there have been contradictory reports in the literature [6,13]. Importantly, studies in cell culture models have established the potential for misfolded WT SOD1 to propagate between cells, leading to the hypothesis that prion-like propagation of misfolded WT SOD1 could be involved in the progressive spread of weakness in sporadic ALS patients [6, 14].

We have previously demonstrated that injecting spinal cord homogenate prepared from paralyzed mutant SOD1 transgenic mice can accelerate motor neuron disease (MND) in transgenic mice expressing the G85R variant of SOD1 [15,17]. To date, most of the work in our laboratory has used a line of mice that express the G85R variant of human SOD1 fused to YFP (G85R-SOD1:YFP) that is largely free of disease until 2024months of age. Injection of tissue homogenates, or purified protein, containing misfolded SOD1 into the spinal cords of newborn mice, or injecting misfolded SOD1 into the sciatic nerve of young adult mice, induces paralysis 215months post-injection [15,17]. Using mice that express untagged human G85R SOD1, Bidhendi and colleagues demonstrated accelerated onset of paralysis in mice when young adult animals were similarly injected with preparations derived from spinal cords of paralyzed mutant SOD1 animals or from human SOD1 ALS cases [18, 19]. We have also observed accelerated onset of paralysis in mice that express untagged versions of two other mutant human SOD1 truncation variants [17], which are among the most unstable variants of mutant SOD1 we have examined [20, 21]. Notably, however, in pilot studies with small numbers of animals that express WT human SOD1, the G37R variant, or the G93A variant of SOD1, we did not observe the same robust accelerated onset of paralysis or induction of more severe inclusion pathology [15].

The present study describes a broader analysis of seeding across a larger panel of SOD1 variants to characterize the influence of primary sequence on the efficacy of seeding to induce motor neuron disease in SOD1 transgenic mice. We examine both isologous and heterologous seeding efficiencies across multiple lines of SOD1 transgenic mice. Our findings confirm that mice expressing WT, G93A, or G37R human SOD1 are relatively resistant to isologous prion-like seeding. Mice expressing the G85R and L126Z variants of fALS were susceptible to both isologous and heterologous seeding by tissue homogenates from paralyzed mice expressing mutant human SOD1. We noted that seeds prepared from mice or rats that express the G37R or H46R variants of slowly progressing ALS were among the least efficient in inducing early paralysis in G85R-SOD1:YFP mice. Notably, we observed that mice expressing human G85R SOD1, or G85R-SOD1:YFP, were resistant to seeding by preparations from spinal cords of paralyzed mice over-expressing mouse SOD1 with the same mutation. Other examples of sequence specificity in seeding implicated an amyloidogenic element bordered by amino acids 3138 as potentially important in the propagation of misfolded SOD1 conformations. Collectively, our studies demonstrate that the primary sequence of SOD1 exerts significant influence over the efficacy of the seed preparation and the susceptibility of the host-recipient in the prion-like propagation of misfolded SOD1 conformations.

## Methods

Mice. All of the transgenic mice expressing human SOD1 variants that were used in this study as recipients of seeding injections have been previously described: G85R-SOD1:YFP and WT-SOD1:YFP mice [22], L126Z Line 45 mice [20], G37R Line 29 mice [23], PrP.G37R Line 110 mice [24], QV103Z hSOD1 Line D14 mice [21], G85R Line 148 mice [25], GurWT mice (B6SJL-Tg(SOD1)2Gur/J; stock no. 002297, Jackson Laboratories) [26], Thy1-G93A Line T3 mice (FVB(Cg)-Tg(Thy1-SOD1*G93A)T3Hgrd/J; stock no. 008230, Jackson Laboratories)[27], and VLE G93A mice (B6SJL-Tg(SOD1*G93A)1Gur/ThpaJ; stock no. 032166, Jackson Laboratories [28]. The G85R-SOD1:YFP, WT-SOD1:YFP, and Thy1-G93A mice were maintained on the FVB/NJ background. The GurWT mice were backcrossed to B6/C3F1 hybrid mice for more than 10 generations and maintained on this background. The VLE G93A mice were maintained on the B6SJL hybrid background. All other lines of mutant SOD1 mice were maintained on a hybrid background of C57Bl/6J and C3H/HeJ, which were the strains used in the initial generation of the mice. For identification of genotype, DNA was extracted from mouse tail biopsies and analyzed by PCR as previously described [20, 24].

Spinal tissues were harvested from transgenic animals in our in-house colonies that became paralyzed. In addition to the mice listed above, the source for tissues from MoG86R Line M1 was FVB-Tg(SOD1*G86R)M1Jwg/J (stock no. 005110, Jackson Laboratories, Bar Harbor ME) [29]. The original source for GurG93A mice was B6SJL-Tg(SOD1*G93A)1Gur/J (stock no. 002726 Jackson Laboratories) [26]. The GurG93A mice were backcrossed to B6/C3F1 hybrid mice for more than 10 generations and maintained on this background in our colony. Spinal tissues from paralyzed H46R rats [30] were a kind gift of Dr. Christine Vande Velde (University of Montreal, CHUM Research Center, Montreal Canada). Spinal tissues from paralyzed G85R mice (Line 148) [25] were a kind gift of Dr. Don Cleveland (University of California San Diego, San Diego CA).

All animals were housed one to five to a cage and maintained on ad libitum food and water with a 14h light and 10h dark cycle.

Preparation of inoculum. Spinal cord tissues were homogenized in PBS creating a 10% homogenate (w/v), containing 1:100 v/v protease inhibitor cocktail (Sigma, St. Louis, MO) as previously described [15]. Tissues were disrupted by sonication 4 times for 20s each, with cooling on ice between bouts of sonication. Homogenates were then clarified by a low-speed spin at~800g for 10min and the supernatants were aliquoted and placed at -80C. In studies involving human tissues, we concentrated 200l of the clarified homogenates by centrifugation in an AirFuge at maximum speed for 20min. The resulting pellet was resuspended in 40l of PBS with the protease inhibitor cocktail and solubilized by pulse sonication as described above.

Recombinant SOD1 purification and fibrillization. Recombinant hSOD1 proteins were expressed and purified as previously described [31]. Fibrillar aggregates of purified SOD1 were generated in 200l solutions containing 50M of protein in 20mM potassium phosphate, pH 7.2 with the addition of 10mM TCEP. The protein solutions were incubated in a 96-well plate with the addition of a Teflon ball (1/8-in diameter) at 37C with constant agitation in a Synergy HT plate reader (BIO-TEK, Winooski, VT). Parallel wells containing the same components with 4M Thioflavin T were monitored by fluorescence measurements every 15min using a ex=440/30 filter to excite and a em=485/20 filter to detect emission using the Gen5 software (v1.10.8). Incubations were halted when maximum fluorescence was achieved. The presence of aggregated SOD1 was confirmed by filter trap assay and electron microscopy as previously described [17].

Seeding Experiments. Newborn mice were injected intraspinally (ISP) as previously described [15]. Briefly, P0 neonatal pups were placed in aluminum foil and surrounded with ice until movement ceased and skin tone became cyanotic (510min). Using a 10l syringe equipped with a 1-inch, 30-gauge needle with 30 bevel, the needle was inserted through the skin at midline, about 5mm from the base of the tail, and then into the vertebral column before 1l of the inoculum was slowly injected. For cerebral ventricular injections, 2l of inoculum was slowly injected into each cerebral ventricle by inserting the needle approximately 2mm into the skull, penetrating the skull near bregma. For bilateral muscle injections, 2l of inoculum was slowly injected into the lower hindlimb targeting the gastrocnemius muscles. The accuracy of these techniques of injection was verified in separate cohorts of newborn mice injected with PBS containing 1% Evans blue dye [32]. After injections, pups were allowed to completely recover on a warming blanket and then returned to the home cage where they were monitored to ensure full mobility and no signs of impairment.

Sciatic nerve injections were performed as previously described [16]. Prior to the injection, mice were injected subcutaneously with 2mg/kg Meloxicam (Norbrook, Overland Park, KS, USA) to relieve pain and the injection site was shaved and sterilized. Mice were anesthetized with isoflurane and a small incision was then made in the skin of the hindlimb before the sciatic nerve was exposed at the popliteal fossa. A 30-gauge needle containing the inoculum was inserted in the sciatic nerve and reciprocated 10 times, which has been shown to greatly enhance the efficiency of prion transport to the spinal cord [33]. Two microliters of homogenate were injected under the perineurium of the sciatic nerve and the incision was then closed with stainless steel clips and cleaned. Following surgery, 2mg/kg Meloxicam was administered at 24 and 48h post-surgery.

Tissue collection. Mice were anesthetized with isoflurane and perfused transcardially with 20ml of PBS. The spinal cord and brain were immediately removed and the brains were bisected sagittally with one hemisphere drop fixed in 4% paraformaldehyde in PBS for 2448h at 4C and the other flash frozen on dry ice. The spinal columns were removed and cut into 4 sections. Cervical and lumbar segments were de-roofed and drop fixed in 4% paraformaldehyde in PBS for 2448h at 4C. The other segments were dissected and flash frozen on dry ice. Frozen tissues were stored at 80 C. Fixed tissues were either embedded in paraffin for sectioning at 7m or immersed in 30% sucrose in PBS, mounted in OCT media (Sakura, The Netherlands) and sectioned to 30m using a cryostat. The sections were placed in a dish containing anti-freeze solution (100mM sodium acetate, 250mM polyvinylpyrrolidone, 40% ethylene glycol) at pH 6.5, and stored at 4C. Sections were then mounted onto slides, air-dried overnight, and cover-slipped in mounting media containing DAPI (Vector, Burlingame, CA).

Neuropathology. For imaging YFP fluorescence in paraffin embedded tissues or from cryostat sections, images were captured by epifluorescence on an Olympus BX60 microscope.

For immunohistochemistry and histochemical staining, we examined paraffin embedded tissues. For immunostaining with C4F6 antibody (Medimabs, Montreal, Quebec, Canada), deparaffinized sections were incubated in 95% formic acid for 10min and then washed in PBS. Sections were incubated in 0.3% H_2_O_2_ in PBS for 20min and then incubated in PBS-T with 3% normal goat serum for 1h before the primary antibody C4F6 was added at a dilution of 1:500 and incubated at 4C overnight. The sections were then incubated with a biotinylated secondary anti-mouse antibody (Vector Laboratories, Burlingame, CA) diluted 1:500 in PBS-T with 3% normal goat serum followed by incubation with the ABC-horseradish peroxidase staining kit (Vector Laboratories). Sections were developed using the DAB staining kit (KPL, Gaithersburg, MD) and counterstained with hematoxylin. Images were captured using an Olympus BX60 microscope.

SOD1 inclusions were also visualized by Campbell-Switzer silver staining using a protocol provided by Dr. Robert Switzer (NeuroScience Associates, Knoxville, TN; Campbell S, Switzer R, Martin T (1987) Alzheimers plaques and tangles: a controlled and enhanced silver staining method. Soc Neurosci Abst 13:678) [34]. A subset of the silver-stained sections was counterstained with hematoxylin and all slides were cover slipped using Thermo Scientific Cytoseal 60 mounting medium. Images were obtained using an Olympus BX60 microscope or an Aperio Scanscope XT image scanner (Aperio, Vista, CA, USA). Images captured by the scanner were digitally cropped to generate final images.

The severity of inclusion pathology revealed by direct visualization of fluorescence or after silver staining was scored by a blinded observer (Additional file 1: data file 1). In the G85R-SOD1:YFP mice inclusions appeared as fluorescent puncta in the neuropil, punctate or fibrillar accumulations in the cell body, or as fiber-like structures in the neuropil due to accumulations within processes (axons or dendrites). In silver stained sections, inclusions appeared as dark structures of various sizes, including small neuropil puncta, fiber-like structures in the neuropil, and accumulations of puncta within cell bodies.

## Results

We have demonstrated previously that intraspinal injection of spinal homogenates from paralyzed mutant SOD1 mice into newborn G85R-SOD1:YFP mice accelerates the onset of paralysis and intraspinal SOD1 inclusion pathology [15, 17]. Importantly, we have previously demonstrated that paralysis in G85R-SOD1:YFP mice is not induced by injection of PBS or spinal tissue homogenates from various types of controls (Additional file 2: Table S1) [17]. The controls tested include homogenates from non-transgenic mice, and from transgenic mice that express mutant human tau or mutant human -Synuclein (Syn), and which develop motor phenotypes. Other controls include spinal homogenates from young asymptomatic G85R-SOD1:YFP mice. Additionally, nontransgenic (NTg) littermates injected with homogenates from paralyzed mutant SOD1 do not develop paralysis or exhibit evidence of inclusion pathology (Additional file 1: Data File 1 NTg data tab). Collectively, these previous studies have established the specificity with which spinal homogenates from paralyzed mutant SOD1 mice induce an accelerated paralysis, and inclusion pathology, when injected into the spinal cords of newborn G85R-SOD1:YFP mice.

### Comparative analysis of seeding efficiency by spinal homogenates from paralyzed G37R and G93A SOD1 mice

**Fig. 1:**
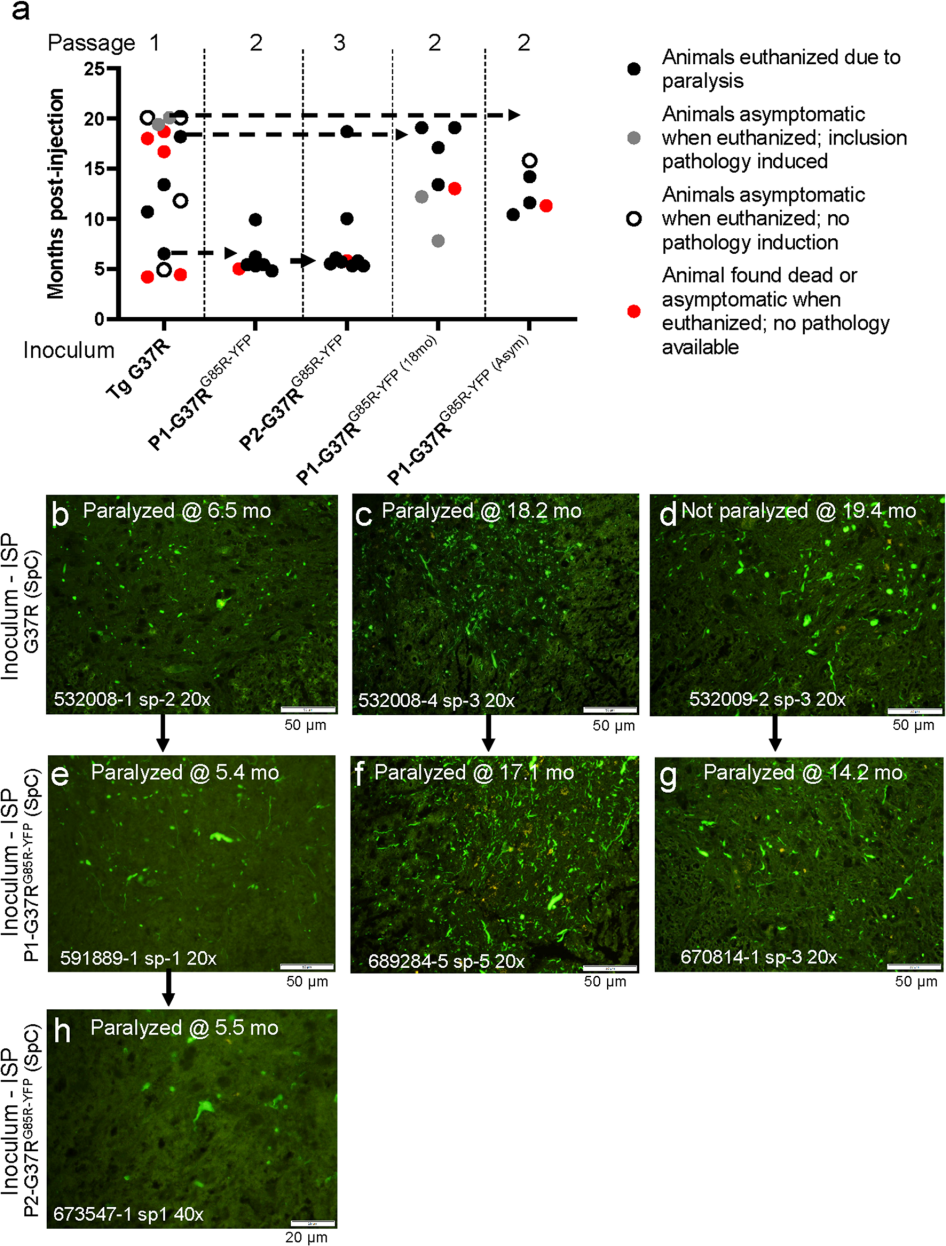
Spinal homogenates from paralyzed G37R SOD1 mice seed G85R-SOD1:YFP mice inefficiently with variable incubation periods. **a** Scatter plot of the ages at which G85R-SOD1:YFP mice developed paralysis or were euthanized after injection of spinal homogenates. Scatter plots in this figure and figures that follow were generated in GraphPad Prism v9. TgG37R denotes spinal homogenate from paralyzed G37R-Line 29 mice. P1-G37R^G85RSOD1:YFP^ denotes inoculum from a paralyzed first-passage G85R-SOD1:YFP animal. P2-G37R^G85RSOD1:YFP^ denotes inoculum prepared from a paralyzed second-passage G85R-SOD1:YFP animal. The dashed lines with arrows mark animals from the initial passage that were used to prepare inoculum for second passage. **bh** Representative images of inclusion pathology in cryostat sections that were induced in the spinal cord of G85R-SOD1:YFP mice that had been injected intraspinally (ISP) with spinal homogenates from G37R mice with subsequent passages (images representative of 23 sections per mouse). The arrows identify pathological specimens that are related due to passage. **bd** Pathology specimens from first passage animals. **eg** Representative pathology specimens from 2nd passage animals. **h** A representative image of pathology specimens from 3^rd^ passage animals. Raw data provided in Additional file 1: Data File 1. Scale bars=50m

In the present study, we have focused on examining the role of the primary sequence of the SOD1 seed and the SOD1 gene expressed by the recipient in the efficiency of prion-like seeding. We have previously reported that spinal homogenates from paralyzed mice expressing the G37R variant, which is associated with disease durations in humans that average 18.7years [35], can seed early paralysis and pathology in G85R-SOD1:YFP mice [15, 17]. Here, we have reanalyzed previously published data, along with additional new data, to assess the variability and efficiency of G37R-SOD1 seeds in G85R-SOD1:YFP mice. Overall, homogenates prepared from paralyzed G37R mice induced early paralysis in only 4 of the 15 injected G85R-SOD1:YFP mice (Fig.1a, black circles). All the mice that developed paralysis also developed inclusion pathology, which appeared as fluorescent puncta in the neuropil with variable levels of accumulation in cell bodies (Fig.1b, c). Additionally, there were accumulations within processes that appeared as fiber-like structures in the neuropil (Fig.1cg). The initial description of the G85R-SOD1:YFP mice demonstrated that this transgene is highly expressed in neurons, with minimal evidence of astrocytic expression [22]. Therefore, it seems likely that most of the inclusion pathology occurs in neurons or neuronal processes. Notably, there were two asymptomatic animals that were euthanized at 19months that exhibited extensive inclusion pathology (Fig.1a gray circles; Fig.1d). Curiously, all 4 of the G85R-SOD1:YFP mice that developed paralysis after seeding were from a single litter. In the subset of mice that developed paralysis, the average age to paralysis was 12.2months, but the range was from 6.5 to 18months (Additional file 1: Data File 1).

In prior studies, we have demonstrated that the efficiency of seeding improved when we passaged G37R seeds from these initial G85R-SOD1:YFP mice to nave recipients [17]. To assess the variability of second passage G37R seeds, we compared second passage data for 3 different isolates. Second passage of seeds from the 19-month-old asymptomatic animal that exhibited inclusion pathology (see Fig.1d) induced paralysis in 3 of 5 injected G85R-SOD1:YFP mice at an average age of 12months post-injection (Fig.1a). Second passage of seeds from an 18-month-old symptomatic animal that exhibited inclusion pathology (Fig.1c) also induced paralysis in 4 of 7 injected recipients at an average age of 17months post-injection (Fig.1a). Second passage of seeds from a 6.5month old symptomatic animal induced paralysis in 6 of 7 injected recipients at an average age of 6.2months post-injection (Fig.1a) [17]. Passage of this isolate for a third time produced paralysis in 7 of 8 recipients at an average of 6.2months post-injection (Fig.1a). All the paralyzed mice in second or third passage experiments exhibited inclusion pathology to varying degrees (Fig.1eh). The level of inclusion pathology seen in the 19-month-old P1 animals that were used to prepare seeds for second passage (Fig.1d) was not obviously different from that of similarly aged paralyzed mice (compare Fig.1bd). Overall, the data are consistent with the idea that the efficiency of seeding by homogenates containing the G37R variant of misfolded SOD1 is relatively low and that seeds imprinted by the G37R conformation can exhibit relatively long incubation periods in second passage.

**Fig. 2:**
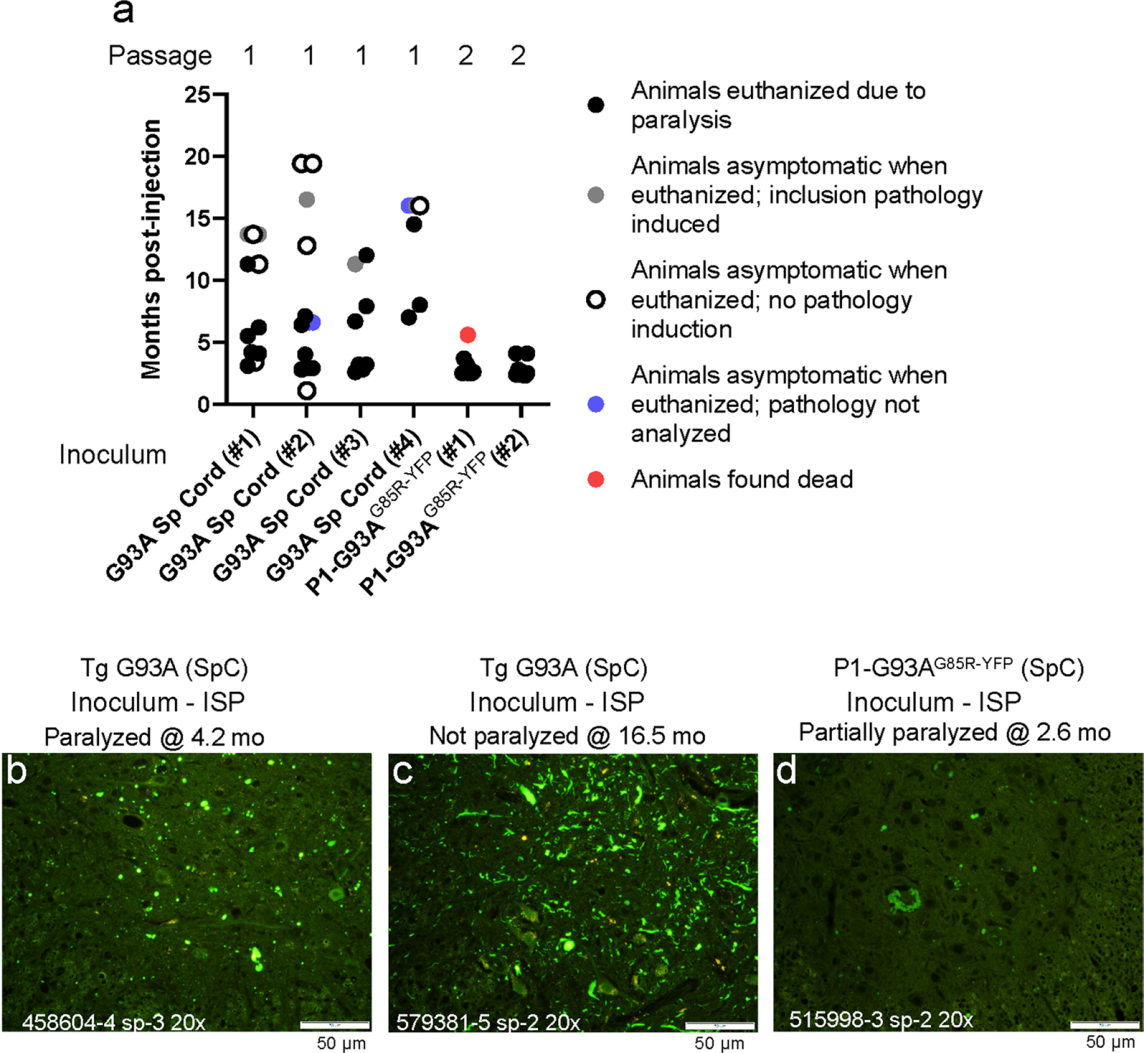
Variability in seeding efficiency of spinal homogenates from paralyzed G93A SOD1 mice in G85R-SOD1:YFP mice. **a** Scatter plot of the ages at which G85R-SOD1:YFP mice developed paralysis or were euthanized after injection of spinal homogenates. TgG93A denotes spinal homogenate from paralyzed GurG93A mice. P1-G93A^G85RSOD1:YFP^ denotes inoculum from a paralyzed first-passage G85R-SOD1:YFP animal. **bd** Representative images of inclusion pathology in cryostat sections induced in the spinal cord of G85R-SOD1:YFP mice that had been injected with spinal homogenates from G93A mice with subsequent passages (images representative of 23 sections per mouse). Raw data provided in Additional file 1: Data File 1. Scale bars=50m

As compared to seeding with spinal homogenates from paralyzed G37R mice, the efficiency of seeding paralysis by homogenates from paralyzed G93A mice was more consistent. In experiments using 4 independently generated homogenates, we observed paralysis in at least half of the injected G85R-SOD1:YFP recipients for each cohort (Fig.2a, black circles). Mice that developed paralysis tended to do so before the age of 12months (Fig.2a). In three of the 4 experiments, the average age to paralysis was 5.7, 4.3, and 4.4 mo post-injection (Additional file 1: Data File 1). In cohort #4, three of the 7 mice that were injected developed paralysis before 16months of age when we terminated the experiment (average age to paralysis was 10 mo post injection) (Fig.2a, G93A Sp Cord #4). Of the 25 total mice that were injected with G93A seeds, 11 lived to 12months post-injection without developing paralysis (Fig.2a; Additional file 1: Data File 1). All mice that developed paralysis exhibited inclusion pathology within the spinal cord (Fig.2b; Additional file 1: Data File 1). Interestingly, as was the case in G37R first-passage mice, we observed several cases in which asymptomatic mice were euthanized at advanced ages (>12months) and found to exhibit extensive inclusion pathology (Fig.2c; Additional file 1: Data File 1). As previously reported [15, 17], second passage of seeds from P1-G93A^G85RSOD1:YFP^ recipients that became paralyzed produced more efficient induction of paralysis at earlier ages (Fig.2a) with inclusion pathology (Fig.2d). The average age to paralysis for second passage G93A seeds was~3months post-injection (Additional file 1: Data File 1). These findings are consistent with the idea that the G93A variant of SOD1 misfolds into a conformation that produces relatively efficient seeds, imprinting conformations to G85R-SOD1:YFP that can produce relatively short incubation periods as seeds in second passage.

### Low seeding efficiency by spinal homogenates from paralyzed H46R-SOD1 transgenic rats

**Fig. 3:**
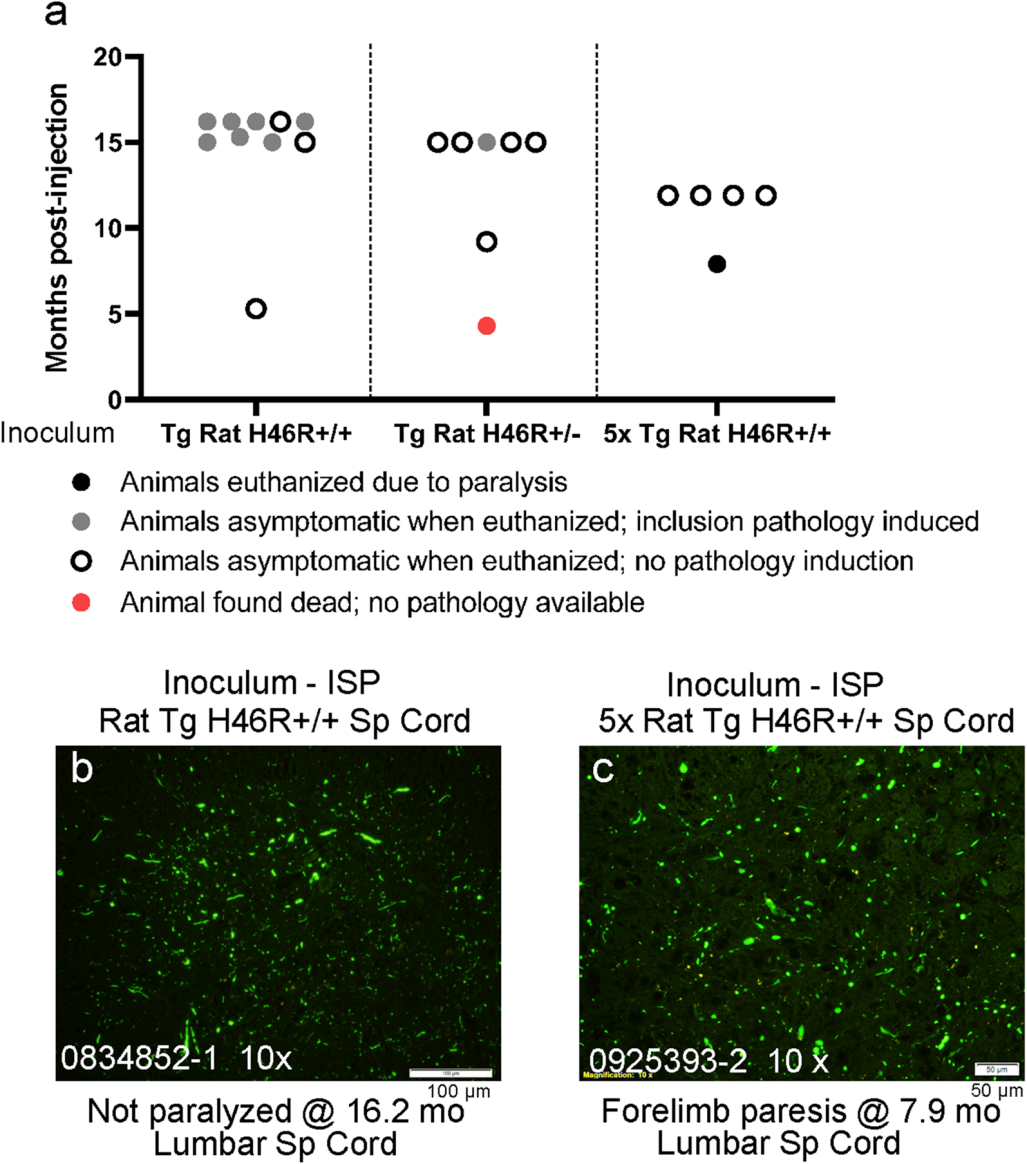
Spinal homogenates from paralyzed H46R rats seed G85R-SOD1:YFP mice inefficiently. **a** Scatter plot of the ages at which G85R-SOD1:YFP mice developed paralysis or were euthanized after injection of spinal homogenates from paralyzed H46R rats. **b**, **c** Representative images of inclusion pathology seen in paraffin sections from the spinal cord of G85R-SOD1:YFP mice that had been injected with spinal homogenates from heterozygous and homozygous paralyzed H46R rats, with a fivefold concentrated inoculum prepared from homogenate from a paralyzed homozygous rat (images representative of 23 sections per mouse). Raw data provided in Additional file 1: Data File 1. Scale bar=100m (**b**) or 50m (**c**)

 The average duration of disease in individuals with the H46R mutation is 17.0years [35]). Spinal cords from paralyzed rats that were heterozygous or homozygous for the transgene (described in [30]) were homogenized and injected into the spinal cords of newborn G85R-SOD1:YFP mice. None of the injected animals developed paralysis and the experiment was terminated at 1516months post-injection to examine pathology (Fig.3a), finding that most of these asymptomatic mice injected with homogenate from the homozygous H46R rats had developed inclusion pathology (Fig.3b). To assess whether a higher dose of H46R seeds may be more effective, we concentrated spinal homogenates from paralyzed homozygous H46R rats by fivefold and injected them into newborn G85R-SOD1:YFP mice. One animal of the 5 that were injected developed forelimb weakness at 7.9months of age, and this animal exhibited inclusion pathology (Fig.3c). The remaining 4 animals from this cohort were asymptomatic when we terminated the experiment at 12months of age, and lacked evidence of inclusion pathology (Fig.3a; Additional file 1: Data File 1). These data suggest that homogenates from paralyzed H46R rats induce paralysis in G85R-SOD1:YFP mice rather inefficiently, despite a capability to induce inclusion pathology.

### Seeding G85R-SOD1:YFP mice with recombinant SOD1 fibrils

**Fig. 4:**
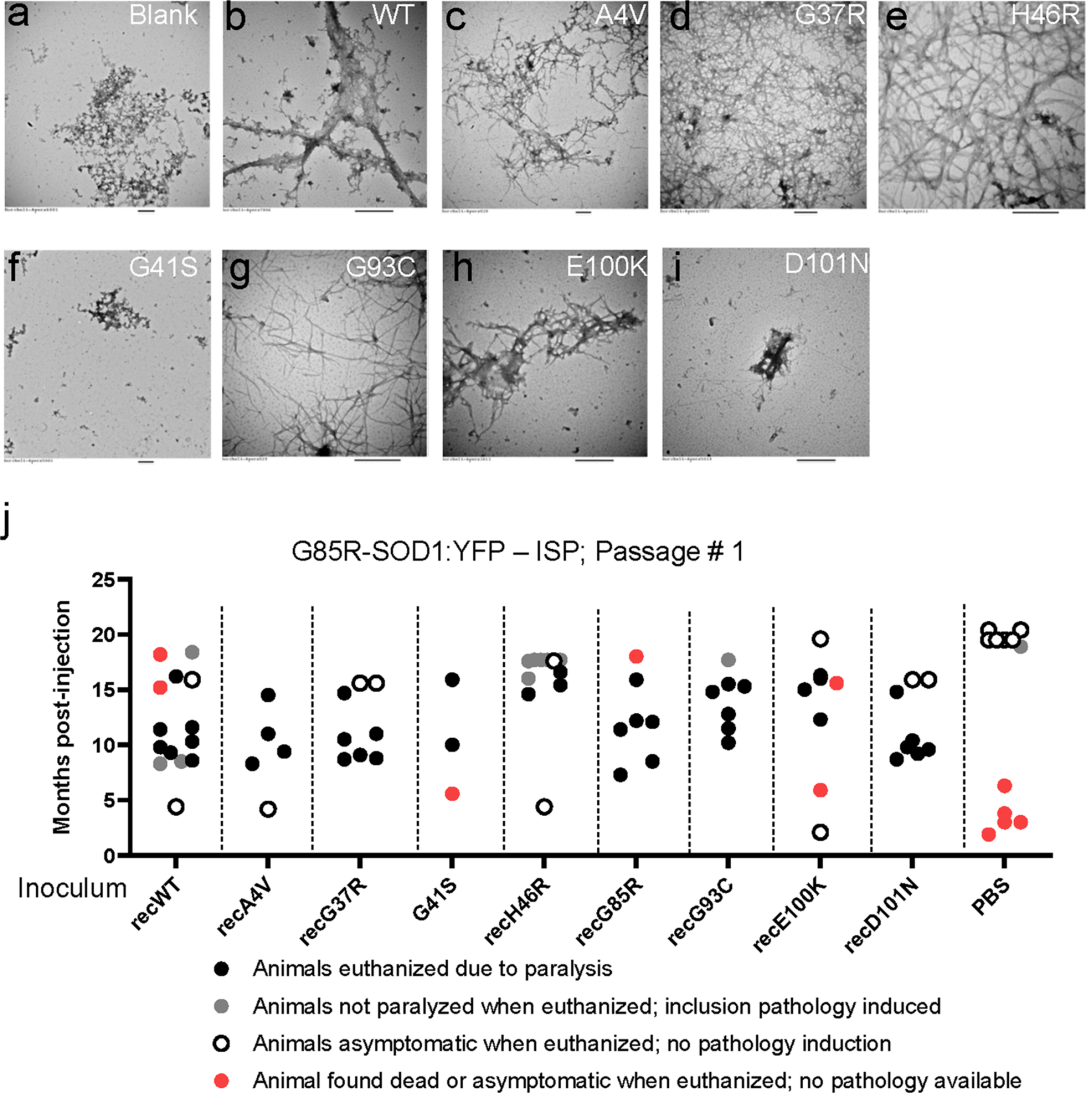
Analysis of the seeding efficiency of recombinant SOD1 fibrils in G85R-SOD1:YFP mice. **ai** Representative images of fibrils formed by recombinant SOD1 aggregated in vitro. **j** Scatter plot of the ages at which the G85R-SOD1:YFP mice developed paralysis or were euthanized after intraspinal injection of recombinant SOD1 fibrils (P0 injection). Raw data provided in Additional file 1: Data File 1. All scale bars (below each panel) represent 500nm

In prior studies, we have demonstrated the induction of paralysis and inclusion pathology in G85R-SOD1:YFP mice by the injection of fibrils produced by recombinant human SOD1 (WT or G93A variants) [17, 36]. To expand the number of different sequence variants we could assess in seeding, we produced recombinant SOD1 encoding eight different fALS mutations and fibrilized these proteins in vitro to produce inoculum for injection (Table 1). To confirm the formation of fibrils, we analyzed the samples both by electron microscopy (EM) (Fig.4ai; Additional file 2: Fig. S1) and by using a cellulose filter trap assay (Table 1). Somewhat surprisingly, over multiple assays, we were unable to detect the generation of fibrillar aggregates by 2 of the 7 mutant SOD1 proteins examined (Table 1; G85R mutant was not examined by EM). For the G41S and D101N mutants, which are associated with rapidly progressing disease, we only observed debris that was similar to what we observed in the control blank (Fig.4a, f, and i; Table 1). Based on prior studies of the H46R variant in vitro [37, 38], we had expected poor fibrilization of this variant in our experiments. However, we were able to produce fibrilized H46R human SOD (Fig.4e). Prior studies have noted that SOD1 fibrilization can be somewhat stochastic and in competition with the formation of amorphous aggregates [39].

**Table 1:** Summary of data for recombinant SOD1 protein seeding experiments

SOD1 protein	Avg survival time in years^a^	Fibrils visible by EM	Induced pathology in G85R-SOD1:YFP slice culture
^b^WT	N/A	Yes	Yes
A4V	1.20.9	Yes	Yes
G37R	18.711.4	Yes	Yes
G41S	0.90.2	No	Yes
H46R	17.07	Yes	Yes
G85R	6.04.5	N.D	Yes
G93C	13.00.4	Yes	Yes
E100K	12.04.1	Yes	Yes
D101N	2.40.9	No	Yes

^a^Data originally reported in[35]

^b^Inclusive of data originally reported in[17]

Due to the uncertainty surrounding the SOD1 species responsible for prion-like seeding in G85R-SOD1:YFP mice, we decided to test all 8 of the SOD1 mutants, using protein that had been treated in a manner to produce fibrilization. Initially, we used a spinal cord slice culture model from the G85R-SOD1:YFP mice to assess pathological seeding activity [17]. We treated the slice cultures with 1l of each of the proteins (50M) and incubated the sections for one month while monitoring for the induction of SOD1-YFP inclusion pathology. All eight of the mutant SOD1 protein preparations were observed to induce the misfolding of G85R-SOD1:YFP in the slice culture model (Table 1; primary data not shown). We then injected the mutant recombinant SOD1 preparations into the spinal cords of newborn G85R-SOD1:YFP mice to assess whether we could induce accelerated paralysis. All preparations injected were capable of inducing early disease with the age to paralysis ranging between 8- and 16-months post-injection (Fig.4j). The efficacy of inducing paralysis across the mutants was similar to WT SOD1 fibrils with the notable exception in that most of the mice injected with fibrilized recombinant H46R-SOD1 reached a pre-determined aging endpoint of 17months without developing symptoms (Fig.4j). Further study in second and third passage experiments with spinal cords of G85R-SOD1:YFP mice seeded with recombinant H46R, and other seeds, may further reveal unique attributes of this variant.

Mice that displayed paralysis from injection of these seeds were found to produce inclusion pathology (Additional file 2: Fig. S2; Additional file 1: Data File 1). The inclusion pathology induced by recombinant WT-SOD1 fibrils was distributed in cell bodies and neuropil (Additional file 2: Fig.2a arrows; also see [17]); whereas, for all of the mutants, the dominant pathology was located in the neuropil, and appeared as fiber-like and punctate structures (Additional file 2: Fig.2b-i). Interestingly, all but one of the 17-month-old asymptomatic mice injected with recombinant H46R fibrils were found to have high levels of inclusion pathology (Fig.4j; Additional file 2: Fig. S2e). Collectively, these findings demonstrate that purified recombinant SOD1 with mutations associated with fALS can produce prion-like seeds that can accelerate paralytic disease in the G85R-SOD1:YFP model. The efficiency of seeding did not obviously relate to whether the injected recombinant protein formed large visible fibrils, or whether the mutant was associated with rapidly or slowly progressing disease. While the behavior of the recombinant H46R-SOD1 seeds was similar to that of spinal homogenates from H46R-SOD1 rats, the efficiency of seeding with recombinant G37R-SOD1 seeds was relatively high with incubation periods that were similar to variants associated with rapidly progressing disease (Fig.4j). Apart from the H46R variant, we did not observe an obvious distinction in the performance of recombinant SOD1 seeds from slowly progressing variants (G37R, G93C, E100K) as compared to seeds from rapidly progressing variants (A4V, G41S, D101N).

### Low seeding efficiency with spinal homogenates from older GurWT mice

**Fig. 5:**
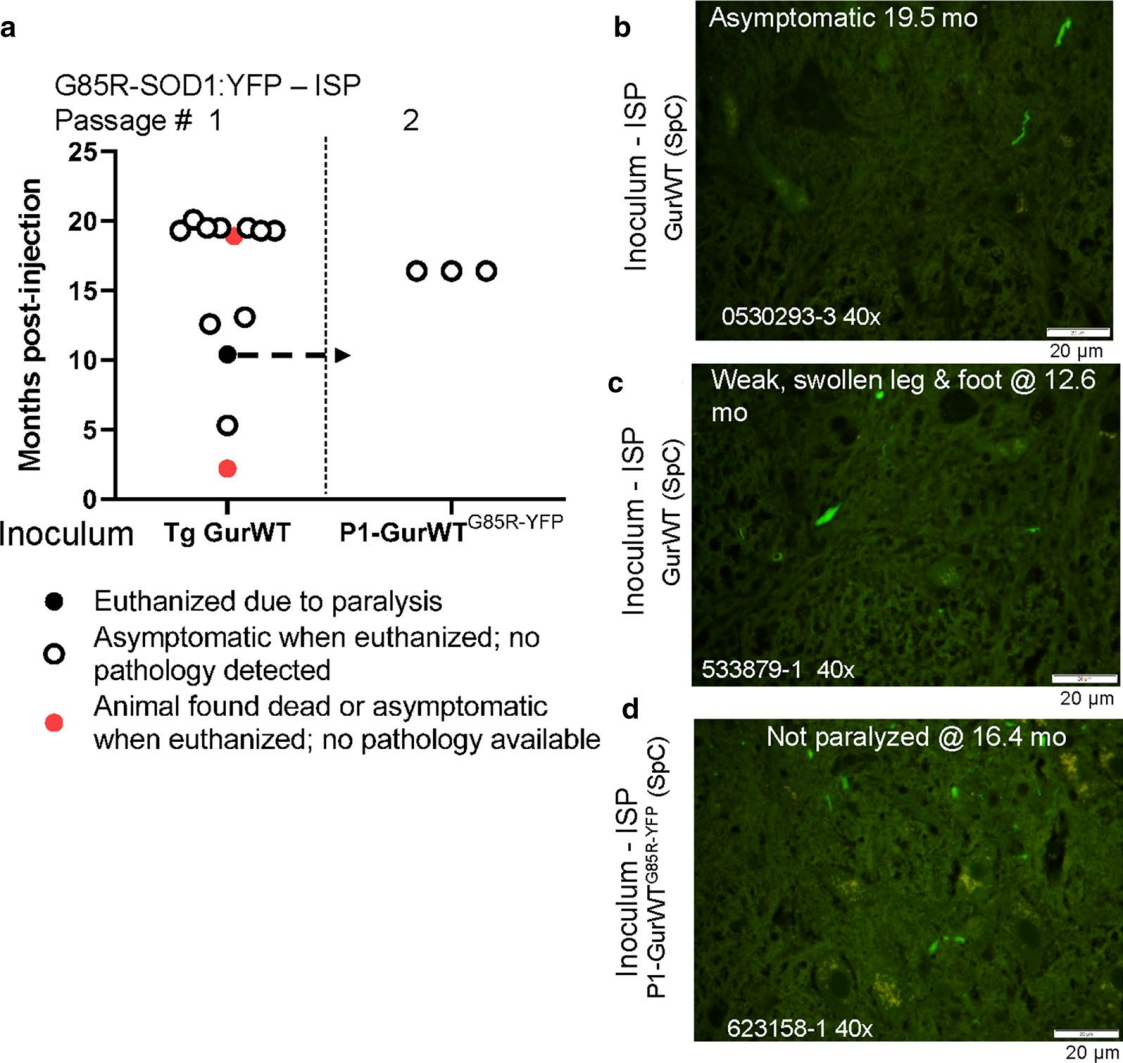
Spinal homogenates from aged GurWT SOD1 mice seed G85R-SOD1:YFP mice inefficiently. **a** Scatter plot of the ages at which G85R-SOD1:YFP mice developed paralysis or were euthanized after injection of spinal homogenates. TgGurWT denotes spinal homogenate from aged GurWT mice. P1-GurWT^G85RSOD1:YFP^ denotes inoculum from a paralyzed first-passage G85R-SOD1:YFP animal that has been previously described [15]. The data graphed here include 3 first-passage animals described in [15]. **bd** Representative images of inclusion pathology seen in cryostat sections from the spinal cord of G85R-SOD1:YFP mice that had been injected with spinal homogenates from aged GurWT mice or with homogenate from the one first-passage animal that developed paralysis (images representative of 23 sections per mouse). Raw data provided in Additional file 1: Data File 1. Scale bars=20m

Previously, we reported that injection of spinal homogenates from older GurWT mice into P0 G85R-SOD1:YFP mice produced paralysis with inclusion pathology in one of the 3 injected animals [15]. One of these 3 injected animals was euthanized at 20months of age with no signs of paralysis or pathology (Fig.5a, b) and the other was found dead at 19months of age with no prior signs of paresis. To further examine the seeding activity of WT-SOD1 in spinal cords of aged GurWT mice, we injected 9 G85R-SOD1:YFP mice. One of these animals developed abnormalities at 12months of age that were described as asymmetrical weakness with swelling in the weak limb and partial paralysis. Despite the suggestion that ALS-like disease was induced, this animal exhibited only sparse fluorescent puncta (Fig.5c). Another animal developed partial paralysis at 13months of age with signs of with injuries to the hindlimbs from fighting or self-mutilation. This animal also showed only sparse fluorescent puncta (Additional file 1: data file 1). To follow up on the one G85R-SOD1:YFP animal that developed paralysis by seeding with GurWT spinal homogenates, we attempted to passage seeds from this animal into nave G85R-SOD1:YFP (P1-WT^G85RSOD1:YFP^). In a small cohort of three mice, none developed paralysis, and when these animals were euthanized at 16.4months of age we once again observed only sparse fluorescent puncta (Fig.5d). These data indicate that the efficiency of seeding paralysis, or inclusion pathology, in G85R-SOD1:YFP mice by injecting spinal cords of older GurWT mice is relatively low.

### Comparison of prion-like propagation to transgenic co-expression of WT and mutant SOD1 with G85R-SOD1:YFP

**Fig. 6:**
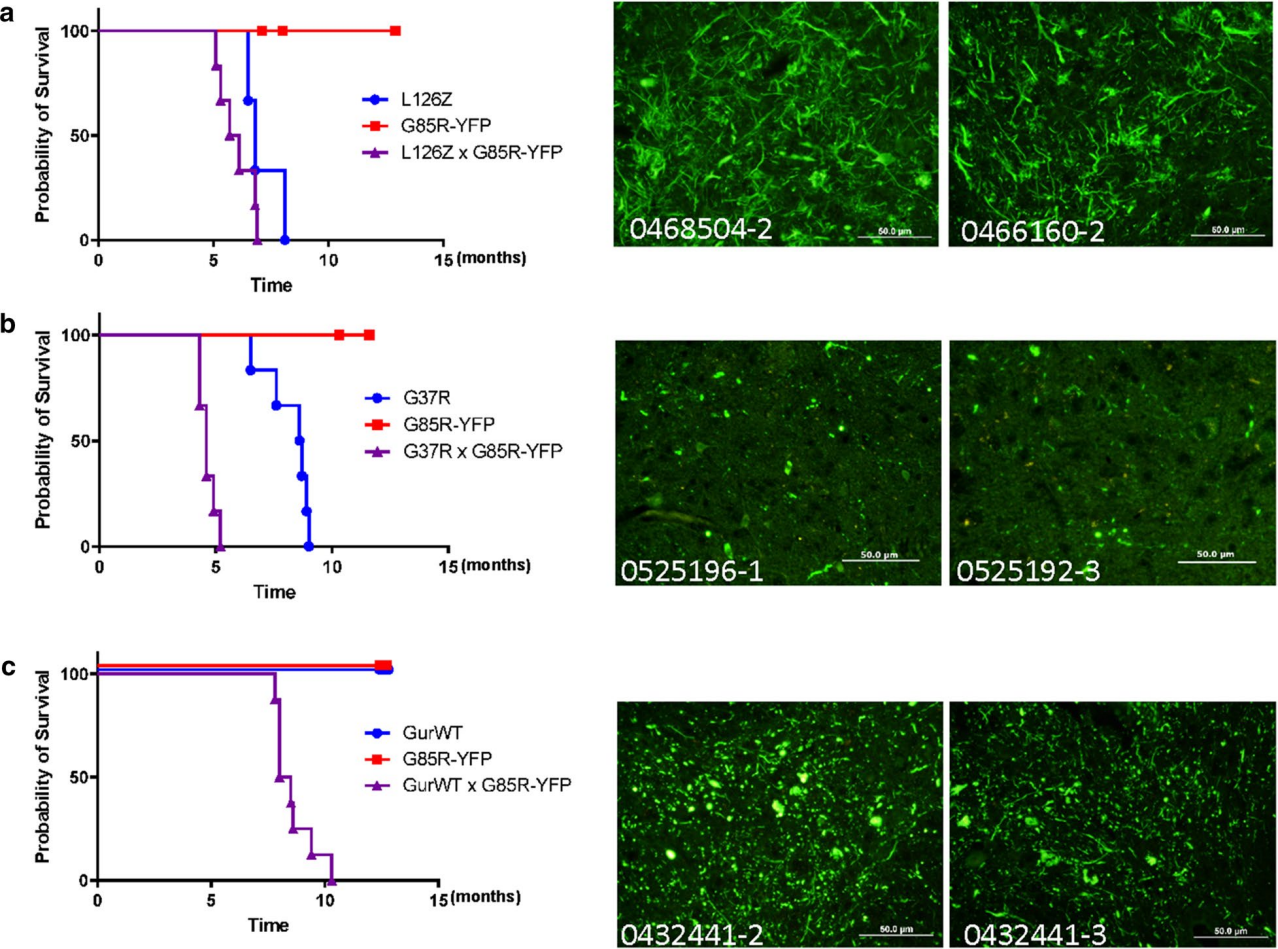
Induction of G85R-SOD1:YFP inclusion pathology by co-expression of untagged mutant SOD1. **ac** KaplanMeier survival curves and representative pathologic images resulting from G85R-SOD1:YFP mice crossed with mice expressing untagged L126Z (Line 45) (L126Z mice n=3, G85R-YFP mice n=5, L45xG85R-YFP mice n=6); untagged G37R (Line 29) (G37R mice n=6, G85R-YFP mice n=10, L29xG85R-YFP mice n=6), or untagged WT human SOD1 (GurWT n=14, G85R-YFP n=5, GurWTxG85R-YFP n=8), respectively. Survival plots in this figure and figures that follow were generated in GraphPad Prism v9. Representative images of inclusion pathology seen in cryostat sections from the spinal cord of paralyzed bigenic mice created in each crossing experiment (images representative of 23 sections per mouse). Scale bars=50m

The co-expression of mutant SOD1 with G85R-SOD1:YFP presents a scenario of sustained high-level exposure of the G85R-SOD1:YFP protein to a misfolded untagged mutant SOD1. We have previously observed that co-expression of human G93A-SOD1 with G85R-SOD1:YFP produced abundant YFP inclusion pathology [15]. This outcome presaged our observation that injection of spinal homogenates from paralyzed G93A mice induced an early paralysis with inclusion pathology in G85R-SOD1:YFP mice [15]. We were therefore interested to examine other mutants in co-expression models. Consistent with L126Z-SOD1 seeding of G85R-SOD1:YFP described previously [17], we found that co-expression of the L126Z-SOD1 variant (Line 45) at levels sufficient to cause disease was able to induce the aggregation of G85R-SOD1:YFP (Fig.6). Notably, the age to paralysis was modestly earlier in L126Z/G85R-SOD1:YFP mice relative to littermates that expressed the L126Z-SOD1 alone (Fig.6a). In the bigenic mice, we observed abundant fibrillar YFP containing inclusion pathology that resembled pathology induced in G85R-SOD1:YFP mice by seeding with spinal homogenates from paralyzed L126Z-SOD1 mice [17]. Bigenic mice co-expressing G37R-SOD1 (Line 29) and G85R-SOD1:YFP developed disease much earlier than mice expressing G37R-SOD1 alone (Fig.6b). Paralyzed bigenic G37R/G85R-SOD1:YFP mice exhibited robust fluorescent inclusion pathology (Fig.6b). Bigenic mice co-expressing WT-SOD1 (GurWT line) with G85R-SOD1:YFP also developed paralysis at early ages with robust inclusion pathology (Fig.6c). The induced pathology was a mixture of fibrillar and punctate structures. Our findings from these crossing experiments would suggest that seeds prepared from older GurWT mice should have been more effective than observed.

### Comparison of injection routes

In a previous study, we have shown that injection of tissue homogenates from paralyzed mutant SOD1 mice into sciatic nerve directly can seed earlier onset of paralysis with intraspinal inclusion pathology [16]. To determine whether we would achieve the same efficiency of seeding by intramuscular injection, we injected the hindlimb muscles of newborn G85R-SOD1:YFP mice with tissue homogenates from paralyzed G93A mice. In this cohort, we observed that all five mice lived to approximately 20months of age without developing paralysis (Additional file 2: Fig. S3). Notably, however, one of these mice developed inclusion pathology and hence there may have been a low level of seed uptake at nerve terminals, resulting in inefficient seeding and reduced penetrance (Additional file 2: Fig. S3).

We next examined the relative efficacy of intracerebral ventricular (ICV) injection of SOD1 seeds into newborn G85R-SOD1:YFP mice. Using spinal homogenates from G85R:YFP mice that had been injected with G93A homogenate (P1-G93A^G85RYFP^ mice) as seeds, we examined the efficacy of the ICV route in inducing paralysis. In a cohort of 6 animals, we noted that the age of paralytic onset was similar to what was observed in ISP injected mice (range of 410months) (Additional file 2: Fig. S3). All paralyzed animals exhibited abundant inclusion pathology. Interestingly, in the ICV seeding paradigm, all the mice first presented with forelimb weakness, suggesting a rostral to caudal spread of the disease-causing conformation.

### Isologous and heterologous seeding in untagged G85R SOD1 mice

**Fig. 7:**
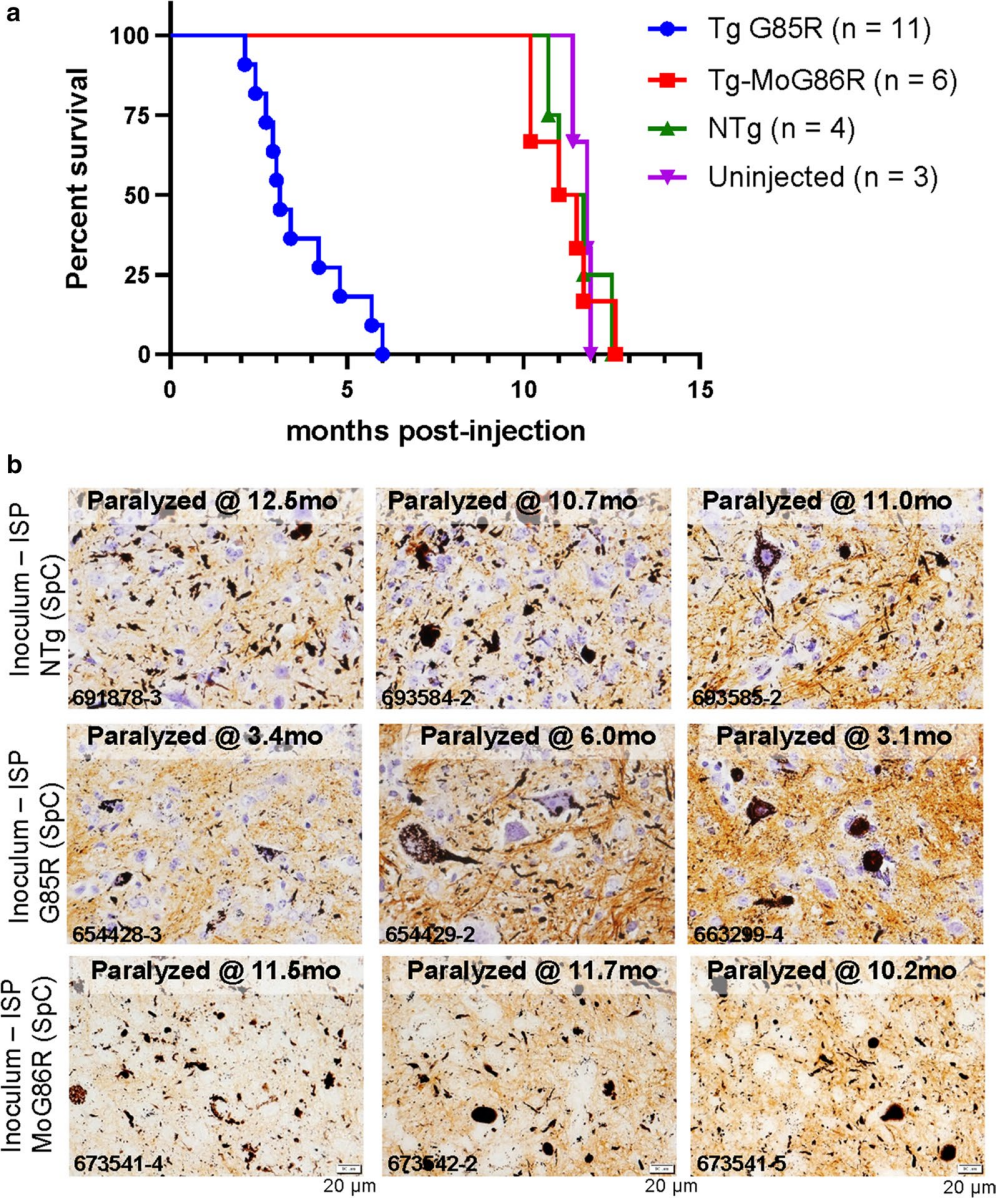
Inefficient seeding of human G85R-SOD1 mice by misfolded murine G86R-SOD1. **a** KaplanMeier survival curves resulting from untagged G85R SOD1 mice injected with spinal homogenates from paralyzed G85R SOD1 mice, paralyzed murine G86R SOD1 mice, NTg mice, or uninjected mice. **b** Representative images of inclusion pathology seen by silver staining of paraffin sections from paralyzed recipient G85R mice (images representative of 23 sections per mouse). Scale bars=20m

As previously reported [19], mice expressing untagged G85R-SOD1 were susceptible to seeding by spinal homogenates prepared from paralyzed G85R-SOD1 mice. The line of G85R-SOD1 mice used here develops paralysis between 11 and 14months of age [25] (Fig.7a). Although there was some variability in the age to paralysis in the seeded G85R mice (range 2.16months), all 11 of the injected G85R mice developed paralysis early (Fig.7a). By contrast, spinal homogenates from mice that express a version of murine Sod1 with the G85R mutation (originally noted as G86R by codon numbering [29]), did not induce earlier paralysis in human G85R-SOD1 mice (Fig.7a). G85R-SOD1 mice injected with spinal cord homogenates from paralyzed MoG86R-Sod1 mice developed paralysis in the same time frame as mice injected with nontransgenic (NTg) homogenates or PBS (Fig.7a). The homogenate from paralyzed MoG86R-Sod1 mice was also ineffective when these seed preparations were injected into G85R-SOD1:YFP mice (Additional file 1: Data File 1; Additional file 2: Fig. S4).

To visualize inclusion pathology in these seeded untagged G85R mice, we used two methods. The inclusion pathology in the G85R mice seeded with spinal homogenates from paralyzed G85R mice was immunoreactive with C4F6 SOD1 antibody as was pathology observed in older G85R mice seeded with NTg homogenates or vehicle control, PBS (Additional file 2: Fig. S5). Due to variation in the performance of this antibody, we switched to a new method of detecting inclusion pathology in untagged SOD1 mice termed Campbell-Switzer silver stain [34], which we have recently found to be specific for mutant SOD1 inclusions [40]. As was seen in sections stained by C4F6, silver staining revealed a mixture of neuropil and cell-body inclusions in all paralyzed G85R mice, regardless of age at which paralysis developed or injection inoculum (Fig.7b). Importantly, mice that became paralyzed early as a result of seeding showed abundant inclusion pathology. These findings indicate that sequence differences between mouse and human SOD1 inhibit the ability of misfolded G85R mouse Sod1 to seed aggregation of human G85R-SOD1.

### Isologous and heterologous seeding in the L126Z-SOD1 and QV103Z-SOD1 mice

**Fig. 8:**
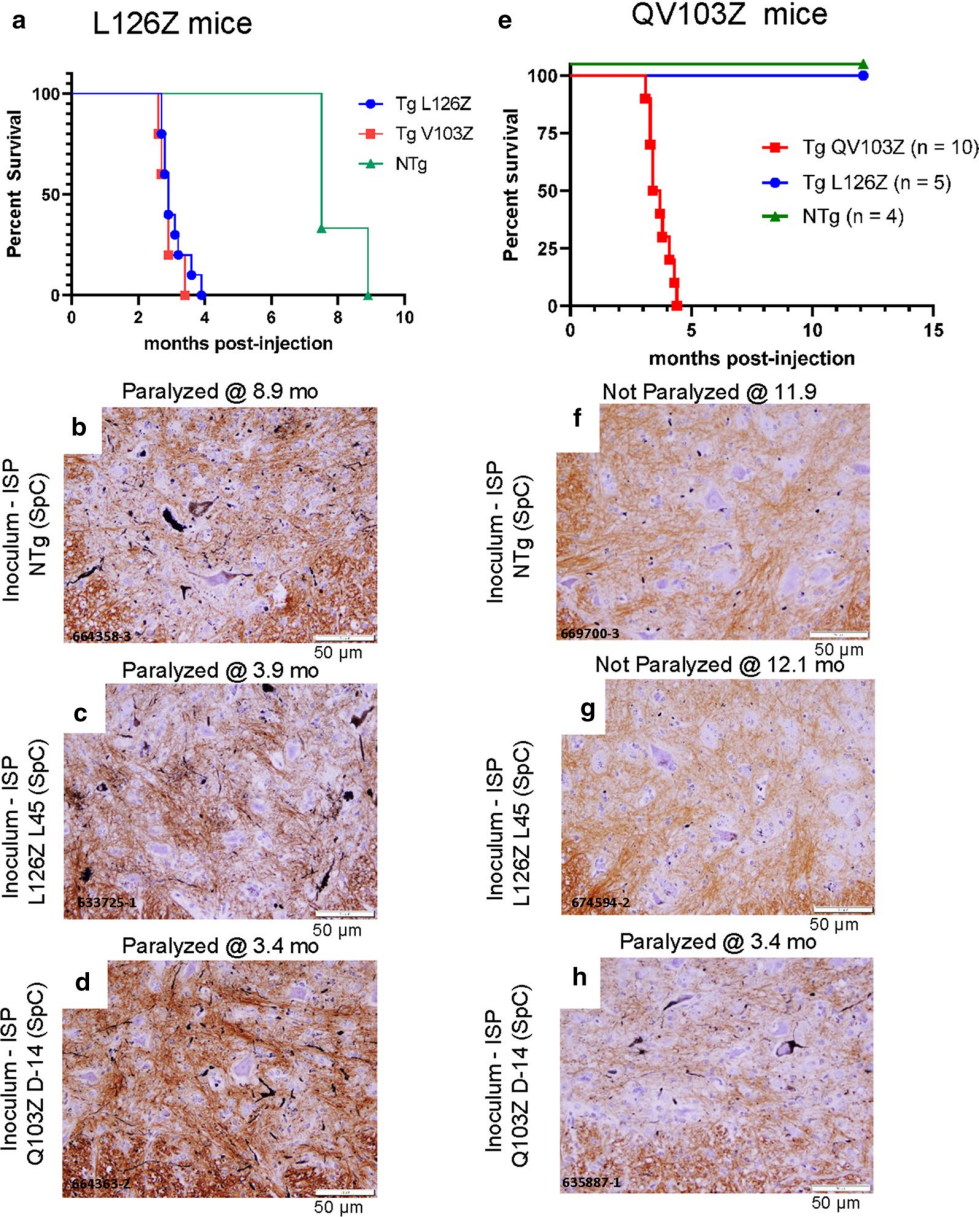
Comparison of the seeding efficiency of L126Z and QV103Z mice in isologous and heterologous seeding. **ad** KaplanMeier survival curves and representative pathology resulting from untagged L126Z SOD1 mice injected with spinal homogenates from paralyzed L126Z or QV103Z mice, and NTg mice. **eh** KaplanMeier survival curves and representative pathology resulting from untagged QV103Z SOD1 mice injected with spinal homogenates from paralyzed L126Z or QV103Z mice, and NTg mice. For each animal 23 sections were examined to identify representative images. Paraffin sections of spinal cord from each animal was stained by Campbell-Switzer method as described in Methods. Scale bars=50m

In prior studies, we have reported that mice expressing untagged mutant SOD1 variants that are truncation mutants (L126Z and QV103Z) show accelerated paralysis after isologous seeding [17]. Both of these lines of mice will develop paralysis on their own, with the L126Z mice developing disease at 79months of age and the QV103Z mice developing paralysis at>14months of age [20, 21]. In subsequent studies of seeding in these lines of mice, we have now found an unexpected distinction in seeding activity. L126Z-SOD1 mice responded to seeding with homogenates from paralyzed L126Z or QV103Z mice (Fig.8a), with the paralyzed mice showing varied levels of silver positive inclusion structures (Fig.8bd; Additional file 2: Fig. S6). By contrast, QV103Z mice were not responsive to seeding by homogenates from L126Z mice (Fig.8e). In silver stains of the asymptomatic QV103Z mice we observed occasional red blood cells (Fig.8f, g), whereas the paralyzed mice seeded with QV103Z homogenates exhibited obvious inclusion pathology (Fig.8h; Additional file 2: Fig. S7). These studies suggest that QV103Z seeds can propagate misfolded conformations to L126Z host SOD1, but when the QV103Z-SOD1 is the host, the conformation carried by L126Z seeds cannot propagate.

### Limited prion-like seeding in mice expressing WT-like mutants of SOD1

We have previously attempted to seed earlier onset disease in Gur1-G93A SOD1 mice G37R-SOD1 mice without success (Additional file 2: Table S2; data from [15]). The GurG93A-SOD1 mice have been reported to have inherently high levels of misfolded SOD1 from very young ages [41], and thus it could be argued that these mice are not responsive to seeding because the pathogenic misfolding of SOD1 is already at saturation. Thus, we turned to lines of mice that express G93A-SOD1 at much lower levels; Thy1-G93A mice (line T3) [27] and a substrain of the G93A mice termed the very low expressing (VLE) G93A mice [28]. Mice from the T3 line that are heterozygous for the transgene do not develop paralysis by 20months of age; whereas, homozygous mice become paralyzed between 15 and 24months of age [27]. Heterozygous Thy-1 G93A mice injected with spinal homogenates from paralyzed GurG93A or G37R-SOD1 mice did not develop paralysis or pathology (Table 2; Additional file 2: Fig. S8). The VLE-G93A mice do not develop paralysis until>22months of age [28]. For these experiments, the source of the seed was spinal homogenates from paralyzed GurG93A-SOD1 (FVB/NJ strain). We used both the newborn intraspinal route of inoculation and the sciatic nerve injection route. For the first time in mice expressing the G93A variant, we observed seeding to produce an earlier onset to paralysis in 6 of the mice injected by the two routes (Table 2). Silver staining confirmed that only the paralyzed mice had inclusion pathology (Additional file 2: Fig. S9). Thus, we demonstrate that VLE-G93A mice are modestly susceptible to isologous seeding with G93A spinal cord homogenates.

**Table 2:** Summary of data from seeding Thy1-G93A Line T3 mice and VLE-G93A

Host	Inoculum (Route)	# inoculated^a^	# paralyzed	Age to paralysis or euthanasia
Thy1-G93A	PBS	2	0	14.8, 16.6 mo
Thy1-G93A	NTg Sp Cord (ISP)	3	0	17.7, 18.2, 19.1 mo
Thy1-G93A	Gur G93A Sp Cord (ISP)	4	0	Terminated at 19 mo
Thy1-G93A	G37R-L29 Sp Cord (ISP)	4	0	Terminated at 18 mo
VLE-G93A	G93A(FVB) Sp Cord (ISP)	8	3	5.3, 8.3, 13.4
VLE-G93A	G93A(FVB) Sp Cord (ScN)	11	3	5.8, 8, 10
VLE-G93A	PBS (ScN)	5	0	Terminated at 12 mo

^a^Mice that were euthanized for non-MND related conditions, such as fight wounds or tumors, before 12months of age were excluded, unless otherwise noted

We also extended our investigations in seeding disease in mice that express the G37R variant under the transcriptional control of the mouse PrP vector (PrP.G37R-SOD1 Line 110) [24]. Mice from Line 110 that are heterozygous for the transgene develop paralysis at 20+months of age, whereas homozygous mice develop paralysis between 8 and 11months of age [24]. In a prior study, we reported that we were not able to accelerate disease in this model by isologous seeding using either an ICV or a direct sciatic nerve injection route [15]. Here, we used our paradigm of newborn intraspinal injection to seed with spinal homogenates from paralyzed homozygous PrP.G37R-SOD1, paralyzed G37R-SOD1 Line 29, paralyzed P1-G37R^G85RYFP^, or paralyzed GurG93A mice. None of these inocula induced paralytic disease (Table 3). In these older heterozygous PrP-G37R mice, we observed some instances of silver-positive inclusion pathology, but the levels of pathology were similar between mice injected with PBS or transgenic spinal homogenates (Additional file 2: Fig. S10). To date, we have not observed accelerated disease in any attempt to seed mice expressing the G37R variant of human SOD1.

**Table 3:** Summary of data from seeding heterozygous PrP.G37R-SOD1 Line 110 mice

Inoculum (ISP)	# inoculated	Age of euthanasia due to age or non-ALS health issues
PBS	5	Terminated at 16 mo
PrP.G37R-SOD1 Line 110 Sp Cord	6	Terminated at 16 mo
G37R-L29 Sp Cord	4	Terminated at 16 mo
P1 G37R^G85RSOD1:YFP^ Sp Cord	7	Terminated at 9 mo
GurG93A Sp Cord	3	Terminated at 17 mo
GurWT Sp Cord	4	Terminated at 16.8 mo

### Mice expressing WT-SOD1 are largely resistant to prion-like seeding

 In addition to our attempts to seed mutant SOD1 mice, we have also examined whether it is possible to induce paralysis in mice that express WT-SOD1 [15]. In a pilot study with a relatively small cohort of mice that express WT-SOD1:YFP, we did not observe induction of paralysis by 913months of age by seeding with spinal homogenates from paralyzed G93A or G37R mice (Additional file 2: Table S3; Data from [15]). This study was hampered by unexpectedly high losses of mice in the cohort due to fighting injuries. We also attempted to induce paralysis by seeding mice that over-express untagged WT-SOD1 (GurWT-SOD1 mice), again observing no induction of paralysis in a small cohort of mice out to 15months of age (Additional file 2: Table S3; Data from [15]). To more rigorously address whether it is possible to induce paralytic disease in mice that over-express WT-SOD1 we first considered what type of misfolded SOD1 seed would potentially be efficacious in mice expressing WT-SOD1.

To determine what type of seed might be efficacious in WT-SOD1 mice, we examined pathology in mice that co-express WT-SOD1:YFP with G93A- and WT-SOD1 (Additional file 2: Fig. S11). We have previously reported that WT-SOD1:YFP is not induced to form inclusion pathology by co-expression of L126Z-SOD1 [21]. Bigenic mice produced from crosses of GurG93A mice and WT-SOD1:YFP mice developed paralysis at about the same time as mice that express only G93A-SOD1 (Additional file 2: Fig. S11a). As compared to mice that express only WT-SOD1:YFP, in paralyzed GurG93A/WT-SOD1:YFP mice we observed redistribution of the YFP fluorescence into fibrillary or punctate inclusion-like pathology (Additional file 2: Fig. S11b & c). Remarkably, a similar type of punctate inclusion-like pathology was observed in very old bigenic mice generated by crosses of GurWT mice and WT-SOD1:YFP mice (Additional file 2: Fig. S11d). We also noted a remarkable level of fluorescent inclusion pathology in the cerebellar dentate (Additional file 2: Fig. S12). Although these mice showed some obvious gait abnormalities, which occur in older GurWT mice [42], these bigenic GurWT/WT-SOD1:YFP mice did not develop the paralytic phenotypes of mice expressing mutant SOD1.

The foregoing data suggested that spinal homogenates from aged GurWT mice or paralyzed G93A mice could potentially seed aggregation and disease in WT-SOD1:YFP mice. Aged GurWT mice accumulate detergent insoluble WT-SOD1 (albeit at lower levels than mutant mice) [15]. We have now examined 15 WT-SOD1:YFP mice injected with spinal homogenates from aged GurWT mice, finding no animals with ALS-like symptoms by 1620months post-injection (Table 3). Additionally, there was not obvious pathological distinction between WT-SOD1:YFP mice injected with GurWT spinal homogenates and mice with PBS or spinal homogenates of NTg mice (Additional file 2: Fig. S13). To further attempt to induce paralytic disease in the WT-SOD1:YFP mice, we injected animals with recombinant WT human SOD1 that had been fibrilized in vitro. No animals developed paralysis by 1620months post-injection, and none showed an obvious induction of WT-SOD1:YFP inclusion pathology at the time of euthanasia (Additional file 2: Fig. S13). Altogether, these findings indicate that WT-SOD1:YFP mice are relatively resistant to developing paralytic disease, or inclusion pathology, by injection with preparations of misfolded WT or mutant SOD1.

In parallel to our effort in the WT-SOD1:YFP mice, we have attempted to induce paralytic disease in the GurWT-SOD1 mice by newborn ISP injection. In a prior study, we reported that by 15months of age, small cohorts (n=4) of GurWT mice injected with spinal homogenates from GurG93A mice had not developed paralysis [15]. We have now extended this effort considerably, testing whether we could induce paralysis by the injection of fibrilized WT-SOD1, or spinal homogenates of aged GurWT mice, or spinal homogenates of paralyzed mice expressing the QV103Z or L126Z mutants. Cohorts of mice were aged to 1720months of age with none of the animals developing any type of paralytic phenotype (Table 4). Additionally, we could not pathologically distinguish mice injected with GurWT spinal homogenates or fibrilized WT SOD1 from mice injected with PBS or NTg spinal homogenates (Table 4; Additional file 2: Fig. S14). Overall, the data show that WT-SOD1 is relatively resistant to prion-like seeding towards induction of motor neuron disease or the type of inclusion pathology present in mice expressing fALS mutant SOD1.

**Table 4:** Summary of data from seeding WT-SOD1:YFP and GurWT-SOD1 mice

Host	Inoculum (ISP)	# inoculated	# paralysis	Age of euthanasia due to age
WT-SOD1:YFP	PBS	3	0	1720 mo (a)
WT-SOD1:YFP	NTg Sp Cord	4	0	20 mo (b)
WT-SOD1:YFP	GurWT Sp Cord	15	0	1920 mo (b)
WT-SOD1:YFP	recWT fibrils	10	0	1620 mo (d)
GurWT	PBS	3	0	20 mo (e)
GurWT	NTg Sp Cord	3	0	20 mo (f)
GurWT	GurWT Sp Cord	6	0	19 mo
GurWT	recWT SOD1 fibrils	7	0	1620 mo
GurWT	GurG93A Sp Cord	4	0	1720 mo
GurWT	L126Z Sp Cord	3	0	1819 mo (g)
GurWT	QV103Z Sp Cord	3	1	1920 mo (h)

(a) Data originally reported in [15]

(b) 1 found dead @ 7 mo. Data originally reported in [15]

(c) 1 found dead @ 4.3 mo, 2 euthanized for non ALS-related issues at 8.7 and 15 mo. Includes data for 5 animals originally reported in [15]

(d) 1 found dead @ 5.4 mo, 2 euthanized for non-ALS-related issues at 7 and 18 mo

(e) 3 found dead @ 4.5 -5.4 mo, 1 euthanized at 3 mo

(f) Data originally reported in [15]

(g) 1 found dead at 18.8 mo no prior clinical signs

(h) 1 animal showed bilateral weakness at 20months when euthanized but lacked inclusion pathology

## Discussion

**Fig. 9:**
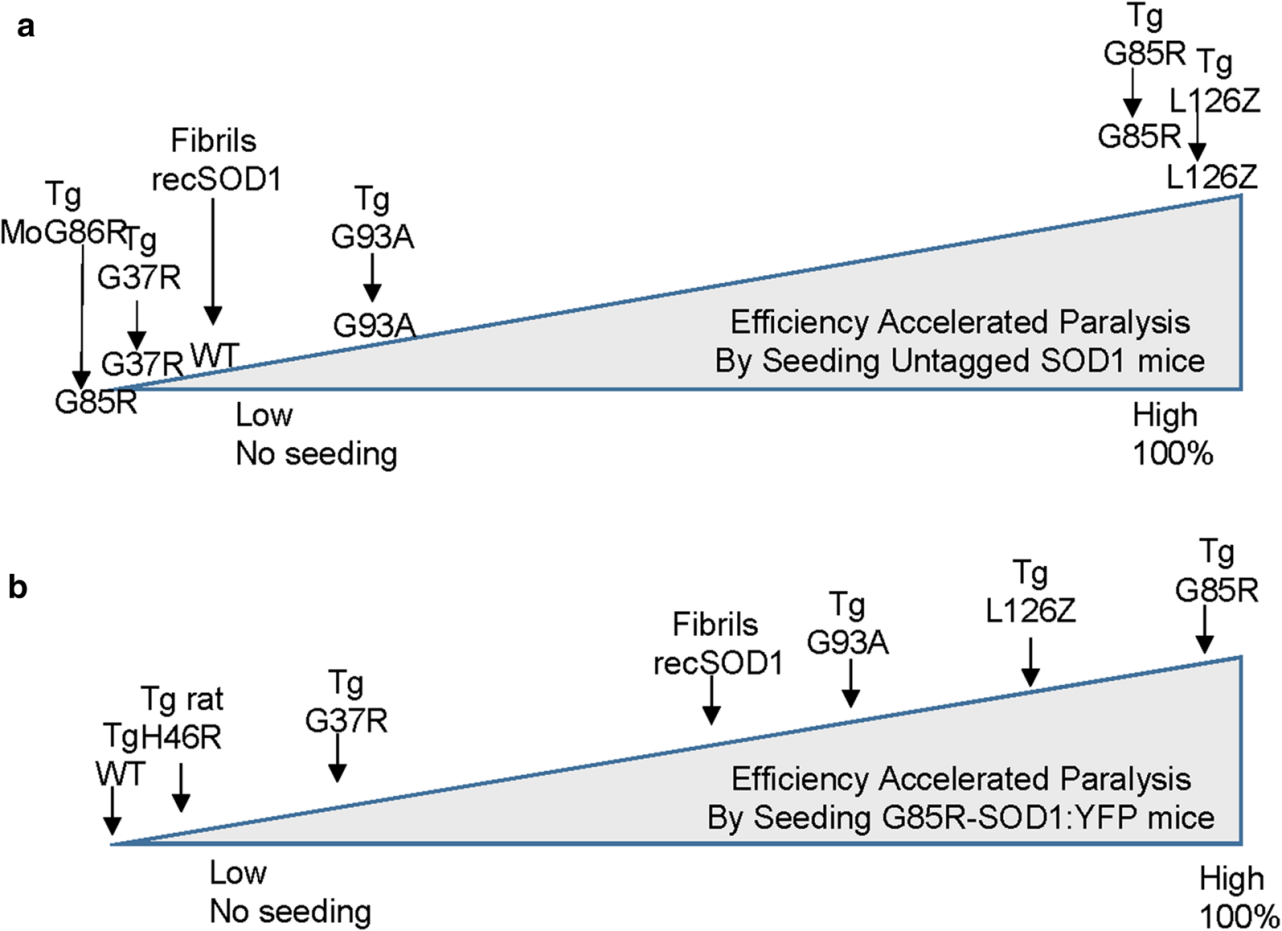
Summary diagrams illustrating the relative efficiency of seeding in mouse models. **a** Summary of findings in seeding experiments involving various lines of SOD1 mice express untagged WT or mutant SOD1. **b** Summary of findings in seeding experiments with G85R-SOD1:YFP mice

The present study builds on prior work to examine the sources of variability in the efficiency of prion-like seeding in mutant SOD1 mice. From the effort we describe here, prior work in our laboratories [15, 17], and data described by Bedhendi [18], it is clear that first passage G85R seeds efficiently seed G85R mice, and that first passage L126Z seeds efficiently seed L126Z mice (Fig.9a). By contrast, mice expressing WT-, G37R-, or G93A-SOD1 variants were partially or fully resistant to isologous seeding (Fig.9a). Notably, mice that express human G85R-SOD1 fused to YFP were found to be susceptible to seeding with a wide range of seeding preparations, including tissue homogenates from paralyzed G37R and G93A mice and preparations of recombinant SOD1 fibrilized in vitro (Fig.9b). In general, heterologous seeding of G85R-SOD1:YFP mice was less efficient than isologous seeding, with seed preparations that contained the G37R or H46R variant being among the least efficient. We further observed that the seeds that arose in G85R-SOD1:YFP mice after seeding by preparations from paralyzed G37R mice appeared to acquire attributes that led to longer incubation times upon second passage. Collectively, these findings show that sequence variation and seed source have profound effects on the prion-like activity of misfolded mutant SOD1.

In assessing the significance of animals that failed to develop paralysis or pathology as a result of intraspinal or sciatic nerve seeding, we must consider the possibility of experimentation error. It is important to note that all of the injections performed in this study were conducted by only two operators that had extensively trained to perform the injections. Although we cannot rule out the possibility of occasional injection error, we have no indication that operator error would explain the data in cases where few or no animals in a cohort developed paralysis. Thus, we conclude that there is considerable variation in the susceptibility of different lines of mutant SOD1 mice to seeding, and that the sequence of the misfolded SOD1 in the seeds can influence prion-like transmissibility to SOD1 transgenic hosts.

### Role of structural stability in the susceptibility of SOD1 variants to seeding

A key distinguishing feature of transgenic SOD1 mice that were vulnerable to seeding versus those that were more resistant could be related to the structural stability of the SOD1 variant expressed by the host. Mice that express WT, G37R, or G93A human SOD1 show high steady-state levels of soluble protein prior to the onset of paralysis, with the accumulation of misfolded SOD1 aggregates occurring as paralysis develops [41, 43, 44]. By contrast, the steady-state levels of soluble mutant SOD1 in G85R mice are relatively low in proportion to transgene mRNA levels [43]. Both the L126Z and QV103Z truncation variants are unstable and show very low steady state levels until they begin to aggregate as paralysis develops [20, 21]. Thus, for the G85R, L126Z, and QV103Z mutants, their susceptibility to isologous, or heterologous, seeding may be linked to poor structural stability.

In WT SOD1 a structurally important intramolecular disulfide bond links Cys 57 to Cys 146 [45]. In studies of SOD1 aggregation and fibrillization in vitro, the presence of the normal intramolecular disulfide bond appears to be a key factor in modulating aggregation [46]. In mutant SOD1 mice that develop paralysis due to over-expression, the misfolded mutant protein that accumulates as pathological inclusions develop lacks the normal intramolecular disulfide bond [43, 44]. Previous analysis of the same G85R mice used here for seeding studies found that all of the mutant protein present in spinal cord of pre-symptomatic mice lacks this critical structural stabilizer [43]. Similarly, the L126Z and QV103Z variants would not be able to produce a normal intramolecular disulfide bond. It is important to note, however, that in GurWT and Gur1G93A SOD1 mice, 814% of the over-expressed protein present in spinal cords of pre-symptomatic mice also lacks the normal disulfide bond [43, 44]. Thus, whether the G85R, L126Z, and QV103Z mice are susceptible to seeding solely because the expressed mutant lacks an intramolecular disulfide bond is unclear.

Another important post-translation modification in the structural stability of SOD1 is the binding of Cu and Zn [47]. Similar to WT SOD1, the G93A or G37R variants, are capable of binding Cu and Zn with high affinity [3, 23, 26]. The G85R variant has been shown to have a weak affinity for Cu and Zn [48, 49] and the enzyme appears to be inactive in vivo [50]. Crystal structures of the G85R SOD1 variant demonstrate disorder in the Zn binding and electrostatic loop elements of SOD1 (amino acids 5083 and 121142, respectively) [48]. The L126Z and experimental QV103Z truncation mutants have not been crystallized, but neither of these variants possess intact electrostatic loop elements. Whether the L126Z truncation mutant can bind Cu or Zn is unknown, but the experimental QV103Z variant that we studied was engineered to remove the critical Cu-binding sites in SOD1 [21]. It is notable, however, that the majority of over-expressed WT or G93A SOD1 present in the spinal cords of Gur1 G93A or Gur WT mice is inactive due to insufficient acquisition of Cu [43]. Thus, the ability of WT and G93A SOD1 to bind Cu does not seem to fully explain the low susceptibility of mice over-expressing these proteins to prion-like seeding if poor Cu binding were the only structural feature involved.

The binding of Zn by nascent SOD1 appears to be critical to achieving a native conformation [51, 52]. Analysis of partially purified homodimeric SOD1 from spinal cords of the GurWT, G37R-Line 29, and Gur1G93A mice found the over-expressed protein appeared have no deficiency in the level of bound Zn; measured to be between 2.5 and 3 atoms per dimer [53]. Thus, it is possible that these variants resist seeding because bound Zn limits prion-like propagation.

Overall, our data indicate that WT SOD1 and WT-like variants are less likely to exist in conformations that are susceptible to prion-like conformational templating as compared to mutants that are more destabilized. Whether any specific structure-stabilizing feature is critical in mediating susceptibility to seeding is unclear. What is clear is that mice expressing the more destabilized variants, G85R, L126Z, and QV103Z were far more susceptible to prion-like seeding. 

### Do ALS-associated mutations in SOD1 imprint strain-like attributes that influence prion-like activity?

Based on our observation that we can induce and monitor the spread of G85R-SOD1:YFP aggregation by seeding into the sciatic nerve of this model, we had begun to link the progressive spread of weakness in SOD1-ALS to the prion-like spread of misfolded conformations [16]. The question that arises is whether there may be conformational attributes that are enciphered by the primary sequence of mutant SOD1 that, by some manner, modulate the ability of misfolded SOD1 to spread within the CNS. It is important to note that in the transgenic mouse models that over-express mutant SOD1, the mutation-specific differences in disease duration that is seen in humans are not recapitulated. For example, mice that express the G93A and G37R variants at similar levels exhibit disease onset and duration that is comparable; 46months to onset and 34weeks duration [23, 26]. Presumably the high-level expression of mutant SOD1 throughout the CNS that is required to produce disease within the lifespan of the animal masks the mutation-specific attribute that causes very slow progression in persons with the G37R mutation. Here, we have asked whether the G37R, or H46R, mutations could impart some specific conformational information to the misfolded SOD1 that accumulates in these animals that would lead to distinct behavior in prion-like transmission studies.

Our newborn injection paradigm is one way to assess the seeding competency of different SOD1 variants. In heterologous seeding in G85R-SOD1:YFP mice by spinal homogenates from paralyzed G93A or L126Z mice (avg. durations of 2.4 and 3.8years in humans, respectively [35]), the efficiency of seeding at first passage was variable. Second passage of G93A seeds through G85R-SOD1:YFP mice, however, produced a consistently earlier onset of paralysis (avg. 2.8months post-injection). Our study of L126Z seeds in G85R-SOD1:YFP mice is more limited, but we did similarly observe consistent acceleration of paralysis by second-passage L126Z seeds in a small cohort of animals (Additional file 2: Fig. S15; data from [17]). Thus, for two mutants associated with rapidly progressing disease we observe relative ease of first passage with accelerated and highly efficient second passage seeding.

For the H46R and G37R variants, associated with slowly progressing ALS in humans, we observed poor seeding activity in first passage, and with G37R-derived seeds we osbserved variable and protracted incubation periods. These data are consistent with the idea that mutations associated with slowly progressing disease may be less efficient in prion-like seeding.

An important consideration in interpreting these results is the potential impact of the host G85R SOD1 variant on any conformational change propagated by exposure to seeds from mice expressing any other variant. In PrP prions, sequence differences between the primary sequence of PrP in seeds and the sequence of PrP in the host can modify strain attributes [54]. Moreover, single amino acid differences of the PrP sequence in the seed and host can create a species barrier that lowers transmission efficiency [55]. To date, G85R-SOD1 mice have shown efficient seeding with spinal cord preparations containing misfolded A4V, G85R, D90A, G93A, and L126 or G127 truncation mutants [15, 17,19]. We observed that fibrilization treatments of 7 different recombinant SOD1 proteins could produce seeds that induce early paralysis in G85R-SOD1:YFP mice. These data indicate that G85R-SOD1 is broadly susceptible to heterologous conformational templating. Indeed, seeds from G37R mice were more effective in G85R SOD1 mice than G37R SOD1 mice. Thus, the poor seeding of G85R-SOD1:YFP mice by spinal tissues from H46R mice and the long incubation periods produced by G37R seeds may indicate that these variants produce strains of misfolded SOD1 that are less effective as seeds to propagate misfolded conformations.

### Is prion-like spreading a common feature in SOD1 ALS?

Our observation that mice expressing the G93A variant are not particularly receptive to seeding would appear to run counter to the notion that prion-like mechanisms of spreading apply to all fALS variants. As indicated above, the G93A mutation is associated with relatively rapidly progressing disease [35, 56], and thus the expectation would be that mice expressing G93A-SOD1 should be highly permissive to seeding, particularly isologous seeding. We have previously reported that the commonly used Gur1-G93A mice did not exhibit accelerated paralysis after intraspinal seeding [15]. To assess whether the level of G93A expression may have been a factor, we tested two additional lines of G93A mice. The Thy-1 G93A mice were unresponsive to seeding; however, with the VLE-G93A mice we began to see evidence that seeding could accelerate the appearance of paralysis albeit at a lower frequency than we would have expected for isologous seeding. As noted above, in the Gur1-G93A mice, a substantial portion of the over-expressed protein lacks Cu and a normal disulfide bond [43]. The absence of these modifications appears to be related to over-expression; increasing Cu loading in Gur1G93A mice dramatically mitigates its misfolding and toxicity [57]. The Cu loading and disulfide status of G93A SOD1 in spinal cords of VLE-G93A mice has not been examined, but the lower expression of mutant protein would be expected to result in lower levels of incompletely modified mutant SOD1.

The absence of seeding efficacy in the Thy-1 G93A mice could potentially be related to the pattern of transgene expression for this model. It is also possible that the level of G93A SOD1 expression in Thy-1 G93A mice is just below threshold to sustain the propagation of misfolded conformations. Additional studies of seeding efficacy in these two lines of G93A mice could provide insight into the basis of vulnerability. At the time of writing, we are not able to explain why the Gur1-G93A or Thy-1 G93A mice did not respond to seeding or why the VLE-G93A showed a modest response. If vulnerability to seeding is by some means related to the folding state of the SOD1 protein in the host, we would have expected the Gur1-G93A mice to be more susceptible to seeding due to a higher level of incompletely modified mutant protein. At face value, the poor seeding of G93A mice could foster skepticism over the role of prion-like spread in the pathogenesis of SOD1-linked fALS; however, there are mitigating factors that merit discussion.

A key variable in our experiments is the level of bioactive misfolded SOD1 seeds in the spinal homogenates we have used for seeding. Previous studies of mutant SOD1 mice that develop paralysis have demonstrated that the levels of misfolded mutant SOD1 in the spinal cords rise steadily and reach maximum when mice are paralyzed [20, 41, 44]. We originally had assumed that at endstage, regardless of age, the levels of misfolded SOD1 in any given animal for a given mutant would be at maximal levels. For all of our mouse to mouse propagation studies, we used 10% homogenates of spinal cord that had been clarified by low speed centrifugation. In our isologous seeding studies of G85R-SOD1:YFP, G85R, and L126Z mice, the levels of the seeds in homogenates from paralyzed transgenic mice were clearly sufficient to induce accelerated paralysis with high efficiency. For the WT-SOD1:YFP, WT-, G93A-, and G37R-SOD1 expressing mice, which did not respond to isologous seeding, it is possible that injection of a higher amount of seeds would have induced disease. Notably, our G93A and G37R seed preparations were effective, to varying degrees, in heterologous seeding of G85R-SOD1:YFP mice. Moreover, recombinant fibrils of WT human SOD1 were highly effective in seeding G85R-SOD1:YFP mice, with no activity in WT or WT-SOD1:YFP mice. Thus, it is clear that the lines of mice expressing WT and WT-like SOD1 mutants (G37R and G93A) are less susceptible to prion-like seeding with preparations that effectively induce early disease in mice expressing the unstable G85R mutant. Whether the lower susceptibility of WT and WT-like variants to seeding could be overcome by injection of higher amounts of misfolded SOD1 seeds will require additional investigation.

An additional consideration in assessing the susceptibility of WT and WT-like SOD1 mutants to prion-like propagation is the timing of exposure to the seed. We have relied heavily on a paradigm in which the seeds were injected into newborn mice, primarily as a means to facilitate wide-spread dissemination of the seeds. The earliest age to paralysis in mice expressing high levels of mutant SOD1 that has been reported is 34months of age [23, 26, 29], suggesting that early in life the toxicity of the mutant SOD1 is suppressed by yet to be defined protective factors. It is possible that mice expressing WT-, G93A-, or G37R-SOD1 do not respond to seeding in the newborn injection paradigm because a combination of protective factors and the natural propensity of these mutants to fold into a more native-like enzyme, suppresses the establishment of a self-sustaining propagative process. It is also possible that the capacity of WT or WT-like SOD1 variants to fold and mature could change with aging as proteostatic protective factors decline. In humans, where disease usually occurs late in life, the WT-like variants of SOD1 could become more prone to adopt conformations that are susceptible to prion-like seeding.

Although our studies, and others [18, 19], demonstrate that mutant SOD1 has the potential to acquire prion-like capabilities, there are aspects of the mouse models that provided sources of misfolded SOD1 seeds that should be considered. In all the mouse or rat models used to produce inoculum, mutant SOD1 is highly expressed throughout the CNS. In this scenario, the development of disease would not necessarily depend on the prion-like spread and therefore there would be little selective pressure towards the accumulation of misfolded SOD1 proteins that can efficiently propagate between cells. It is possible that the absence of such selective pressure leads to the accumulation of mixtures of misfolded SOD1 strains in the transgenic mice that were used to produce the seeds. Any given individual animal could potentially harbor a unique mixture of strains and as such could account for some of the variability we observe in first-passage seeding experiments.

Intriguingly, we observed multiple examples in which seeded mice reached relatively old ages without developing symptoms despite significant inclusion pathology burden. The most striking example was in the cohort of G85R-SOD1:YFP mice seeded with spinal homogenates from paralyzed H46R rats. We also observed high levels of inclusion pathology in aged bigenic mice created by crossing GurWT mice with WT-SOD1:YFP mice. These intriguing findings raise the possibility that some conformations of misfolded SOD1 may be non-toxic. Additional studies are required to understand whether strain variants of misfolded mutant SOD1 may exist that propagate between cells more slowly, or produce less toxic aggregates, and whether such strain variations explain aspects of human SOD1 linked ALS.

### Role of WT-SOD1 in sporadic ALS

The role of prion-like propagation in sporadic ALS, and the identity of the misfolded propagon, remains to be determined. Multiple studies have examined whether a misfolded form of WT-SOD1 could be propagating throughout the neuraxis in sporadic ALS patients (reviewed in [58]). Recombinant WT-SOD1 that has been fibrilized in vitro clearly possesses high seeding activity when injected into G85R-SOD1:YFP mice [17], but these same preparations showed no activity in the WT-SOD1:YFP or GurWT mice. From our experiments in which mutant SOD1 was co-expressed with WT-SOD1:YFP, it was clear that with sustained exposure it is possible to induce WT-SOD1:YFP to produce inclusions. Collectively, our data indicate that although it is possible to create a seeding-competent conformer of WT-SOD1, it does not appear to transmit to WT substrates efficiently in short-term seeding paradigms. It is possible that propagation of misfolded conformations by WT-SOD1 is inhibited by the high propensity of WT-SOD1 to acquire a stable native conformation. Whether other types of conformational changes in WT-SOD1 that could contribute to ALS, and possibly propagate in a prion-like fashion as suggested by other studies [10, 58, 59], has not been ruled out by our study. However, we have not observed WT SOD1 to be able to support propagation of a disease-causing misfolded SOD1 conformation thus far.

### Potential role of amyloidogenic segments in prion-like propagation of misfolded SOD1

Although the G85R-SOD1:YFP, or untagged G85R, mice appear to be highly susceptible to seeding, we observed that seeds prepared from mice that over-express murine SOD1 with the G85R mutation were not effective in either host. Incompatibility between the misfolded seed and the host was also noted when QV103Z mice were seeded with spinal homogenates from L126Z mice, but not vice versa. These seemingly disparate outcomes may provide clues to initial sites of interaction between the propagating seed and the nave host protein. Ivanova and colleagues identified four amino acid segments in SOD1 that were highly prone to aggregation [60]. The four segments were distributed across the protein at residues 1421, 3038, 101107, and 147153. The ability of misfolded QV103Z to propagate to itself or L126Z-SOD1 indicates that if these segments are involved in prion-like propagation, then the segments 1421 and 3038 must be involved. For the L126Z mutant, segment 101107 could also be important because although L126Z could self-seed, it could not cross seed to QV103Z. It is also noteworthy that mice expressing the G37R variant are largely resistant to seeding, and similarly, a synthetic peptide of the amyloidogenic segment 3038 with the G37R mutation was much slower to aggregate in vitro [60]. Segment 3038 is also of interest because of significant sequence divergence between mouse and human SOD1 within this segment; the human sequence is KVWGSIKGL and the mouse sequence is VLSGQITGL. Our studies clearly show that misfolded mouse G85R-SOD1 seeds are not transmissible to mice expressing human G85R-SOD1. In segments 1421 and 101107 there is only one amino acid difference between mouse and human SOD1, and in segment 147153 there are no differences. One of the differences between human and mouse sequence in segment 3038 is a W to S change at position 32, which has been implicated in prion-like propagation of misfolded SOD1 [14, 36, 61, 62]. Collectively, these data suggest the amyloidogenic segment between residues 3038 could be an important modulator of prion-like propagation of misfolded SOD1.

## Conclusions

The collective data of the present study and prior work [15,17, 19] clearly show that spinal cords of paralyzed mutant SOD1 mice contain seed-competent forms of misfolded SOD1 that can induce a self-propagating process, leading to accelerated motor neuron disease and inclusion pathology. Similar seeds are found in human spinal tissues from SOD1-linked ALS patients [19]. Overall, our findings generally align with the hypothesis that prion-like propagation of misfolded conformations could mediate the progressive spread of weakness from limb to limb that is a defining feature of the disease. Tissue homogenates from mice that express mutations associated with more rapidly progressing ALS (G93A, G85R, L126Z) were generally more effective in seeding than homogenates prepared from mice expressing mutants associated with slowly progressing disease (G37R, H46R). Mice expressing the G85R and L126Z mutants were more permissive to seeding than mice expressing the G37R variant. At face value, the inability to efficiently seed G93A mice weakens the idea that prion-like propagation mediates the perceived spread of weakness in all cases of SOD1-linked ALS. Additional studies are required to understand the poor response of mice expressing the G93A variant to seeding; perhaps it is a question of timing or dose of the seed. Our study suggests that further investigations of the prion-like propagation of misfolded conformations in mutant SOD1 could reveal mechanisms that underlie disparate durations of illness in individuals with different mutations in the same gene.

## Supplementary Information

**Additional file 1**. Excel spreadsheet that lists all of the transgenic animals, and a subset of the nontransgenic animals, used in this study, with details on the inoculum injected and incubation period postinjection. See Supplementary Materials for additional information.

**Additional file 2**. This file contains all Supplementary Material, including Supplementary Tables S1-S3 and Supplementary Figures S1-S15.

## Data Availability

Any data generated or analyzed during this study that are not included in this published article and its supplementary files, are available from the corresponding author on reasonable request.
