# Supplementary material for: Variation in the vulnerability of mice expressing human superoxide dismutase 1 to prion-like seeding: a study of the influence of primary amino acid sequence
Source: Acta Neuropathol Commun. 2021 May 20;9:92. doi: 10.1186/s40478-021-01191-w (PMC8139116; doi:10.1186/s40478-021-01191-w)
Supplement: Supplementary file 2 — Additional file 2. This file contains all Supplementary Material, including Supplementary Tables S1-S3 and Supplementary Figures S1-S15. [file 40478_2021_1191_MOESM2_ESM.pdf]

Electronic Supplementary Material: Additional File 2

Variation in the vulnerability of mice expressing human superoxide dismutase 1 to prion-like seeding; a study of the influence of primary amino acid sequence

Jacob I. Ayers[1,2,3], Guilian Xu[1], Kristy Dillon[1], Qing Lu[1], Zhijuan Chen[1], John Beckman[4], Alma K. Moreno-Romero[4], Diana L. Zamora[4], Ahmad Galaleldeen[4], and David R. Borchelt[1,5]

[1]Department of Neuroscience, Center for Translational Research in Neurodegenerative Disease, University of Florida, Gainesville, Florida, USA 32610.

[2]Institute for Neurodegenerative Disease, Weill Institute for Neurosciences, University of California, San Francisco, California, USA 94143.

[3]Department of Neurology, Weill Institute for Neurosciences, University of California, San Francisco, San Francisco, CA 94143

[4]Department of Biological Sciences, St. Mary's University, San Antonio, TX 78228

[5]SantaFe HealthCare Alzheimer's Disease Research Center, McKnight Brain Institute, University of Florida, Gainesville, Florida, USA 32610

Additional Data File 1: The excel spreadsheets list all transgenic animals, and a subset of the non-transgenic animals, used in this study, with details on the inoculum injected and incubation period post-injection. Brief notes on tissue collection methods, clinical appearance, and an assessment of inclusion pathology are also provided. Inclusion pathology scores were conducted by a blinded observer using the following guides; (-) no inclusion pathology noted on any slide; (+/-) 1 or more inclusions present but some sections lack inclusions; (+) 1-5 inclusions present on all sections examined; (++) numerous inclusions on all sections; (+++) inclusions on all sections – too numerous to count. The animal identification numbers in the spread sheet are also indicated on individual images in the main and supplementary figures.

Table S1. Summary of previously reported control seeding data in G85R-SOD1:YFP mice [17].

Inoculum Route - ISP	Passage	# injected[a]	# paralyzed	Age range (avg age paralysis)	# with G85R-SOD1:YFP inclusions
PBS	1	7	0	19.4-20.4	0[b]
NTg homog.	1	6	0	15-20.1	0[c]
Asym. G85R-SOD1:YFP Sp Cord	1	11	0	12-19.2	1[d]
Paralyzed M83 Sp Cord - alpha-syn	1	4	0	12.5 – 16.2	0[e]
Paralyzed JNPL3 Sp Cord - tau	1	7	0	15.9-16.3	0[f]

[a]Mice that were euthanized for non-MND related conditions before 12 months of age were excluded, unless otherwise noted.
[b]Six additional animals that were injected with PBS were euthanized early for injuries or tissue collection, and were not paralyzed at the time of euthanasia. Data originally reported [17].
[c]Four additional animals that were injected were euthanized early for injuries or tissue collection, and were not paralyzed at the time of euthanasia. Data originally reported [17].
[d]Six G58R-SOD1:YFP mice that were injected with spinal homogenates from asymptomatic G85R-SOD1:YFP mice were asymptomatic when harvested at 12 months post-injection. Data on 5 animals originally reported in [17].
[e]Data originally reported [17].
[f]Two animals reported as paralyzed – no pathology available. One animal found dead – no pathology available. Data originally reported [17]

Table S2. Summary of previously reported seeding experiments in GurG93A-SOD1 and G37R-SOD1 mice [15].

**GurG93A P0 ISP injections of spinal homogenates**		
Inoculum	# accelerated/ # inoculated	Age of euthanasia due to paralysis in months
Uninjected	0/4	4.9, 5.1, 5.3, 6.0
PBS	0/3	5.1, 6.5, 6.5
NTg sp cord	0/7	5.1, 5.1, 5.7, 6.1, 6.1, 6.4, 6.8
G93A sp cord	0/6	5.0, 5.0, 5.4, 5.5, 5.6, 5.7
G37R-L29 sp cord	0/6	4.4, 4.9, 5.1, 5.7, 6.4
**G37R-Line 29 P0 ISP injections of spinal homogenates**		
Uninjected	0/3	8.1, 8.1, 8.1
NTg sp cord	0/4	8.7, 8.7, 8.7, 8.8
G37R-L29 sp cord	0/7	7.0, 7.4, 8.0, 8.2, 8.3, 8.4, 8.9
G93A sp cord	2/5	5.6, 5.7, 6.5, 7.8, 8.3

Table S3. Summary of previously reported seeding experiments in WT-SOD1:YFP and GurWT-SOD1 mice [15].

**WT-SOD1:YFP P0 ISP injections of spinal homogenates**		
Inoculum	# inoculated	Age of euthanasia due to age or non-ALS health issue in months
PBS	4	3 mice originally reported disease free at 15.1; Terminated at 17-20 mo asymptomatic
NTg sp cord	4	3 mice originally reported disease free at 15.2; Terminated at 20 mo asymptomatic
GurWT sp cord	5	Originally reported disease free at 12.7; Terminated at 20 mo asymptomatic
G37R-L29 sp cord	4	3 of 4 mice were lost due to injury or early death; One animal originally reported disease freed at 13.6 – terminated at 20 mo asymptomatic
GurG93A sp cord	6	4 of 6 mice were lost due to injury or early death; Two animals originally reported disease free at 9.2 – terminated at 19.5 mo asymptomatic
**GurWT P0 ISP injections of spinal homogenates**		
PBS	4	Originally reported disease free at 15.1 – terminated at 20 mo asymptomatic
NTg sp cord	3	Originally reported disease free at 15.2 – terminated at 20 mo asymptomatic
G93A sp cord	4	Originally reported disease free at 15.4 – terminated at 17-20 mo asymptomatic

Supplemental Figure S1

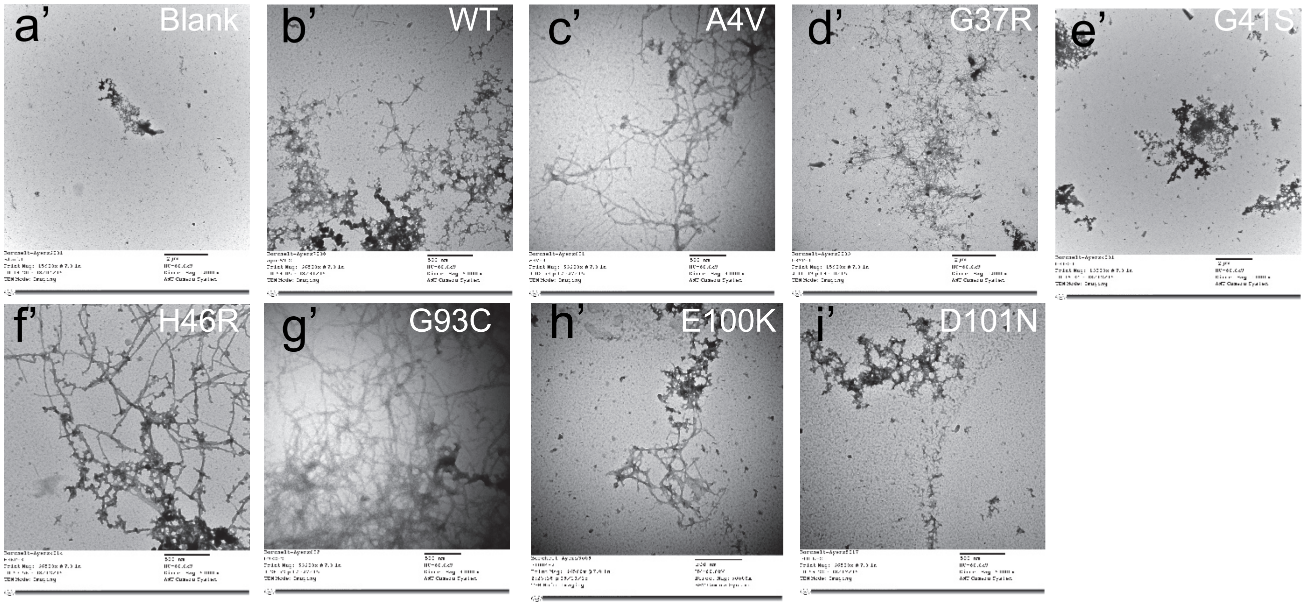

Fig. S1 Representative images of fibrils formed by recombinant SOD1 aggregated *in vitro*. A second set of images for each of the mutants examined. Fibrillar structures were clearly visible in preparations of WT, A4V, G37R, H46R, G93C and E100K recombinant human SOD1. In preparations of G41S and D101N, the material that was visible resembled debris that could be found in the control blank. Scale bars = 500 nm

Supplemental Figure S2

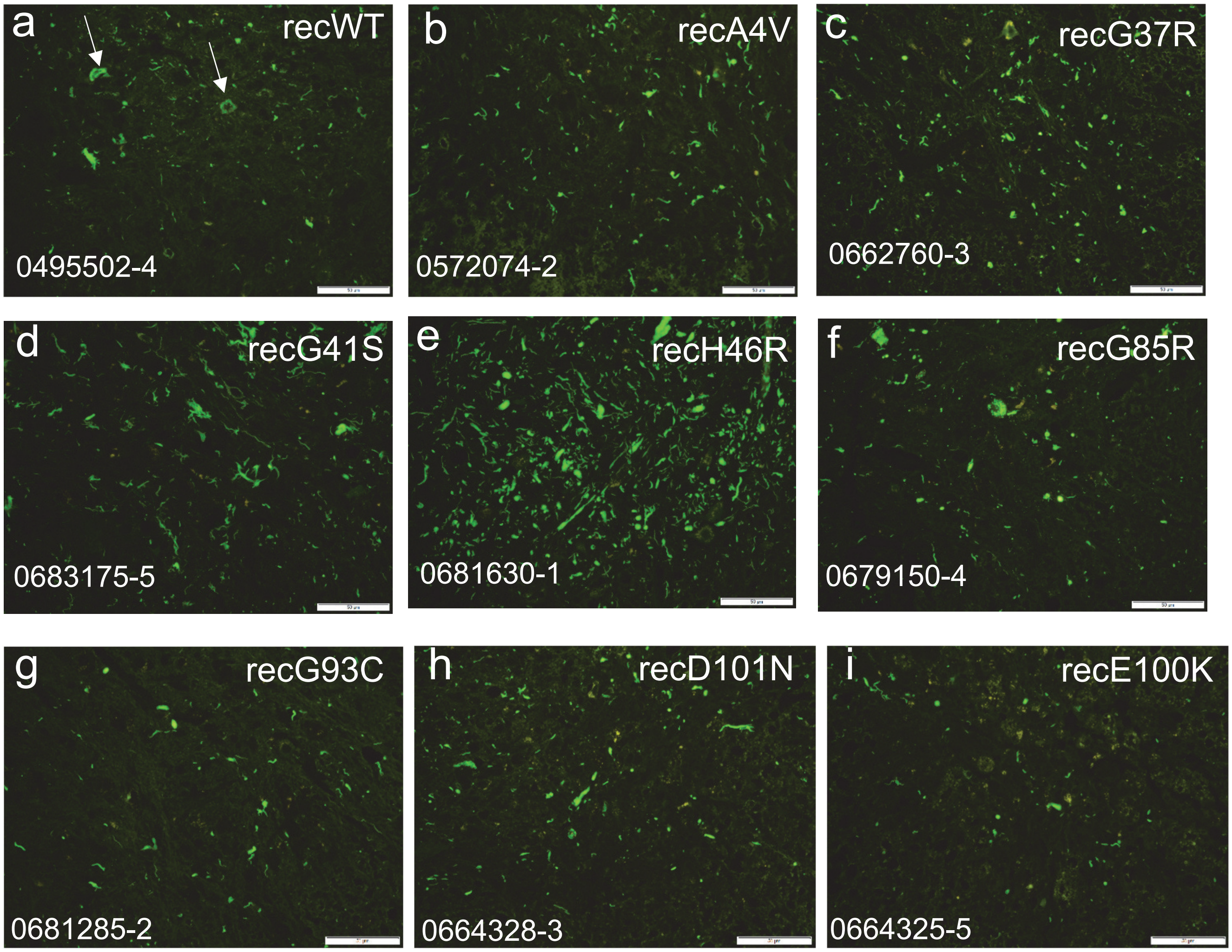

Fig. S2 Representative images of inclusion pathology induced in the spinal cord of G85R-SOD1:YFP mice that had been injected with recombinant SOD1 fibrilized *in vitro*. YFP fluorescence images were captured as described in Methods. The images shown are representative of the ventral horn of the lumbar or cervical spinal cord (2-3 sections per animal were visualized). For these studies, YFP fluorescence was imaged in paraffin sections. The total number of animals examined and pathologically scored is documented in Supplemental Data File 1. Images representative of 2-3 sections per mouse. Scale bars = 50 µm. The arrows in panel (a) mark cell bodies that appear to be filled with fibrillar inclusions.

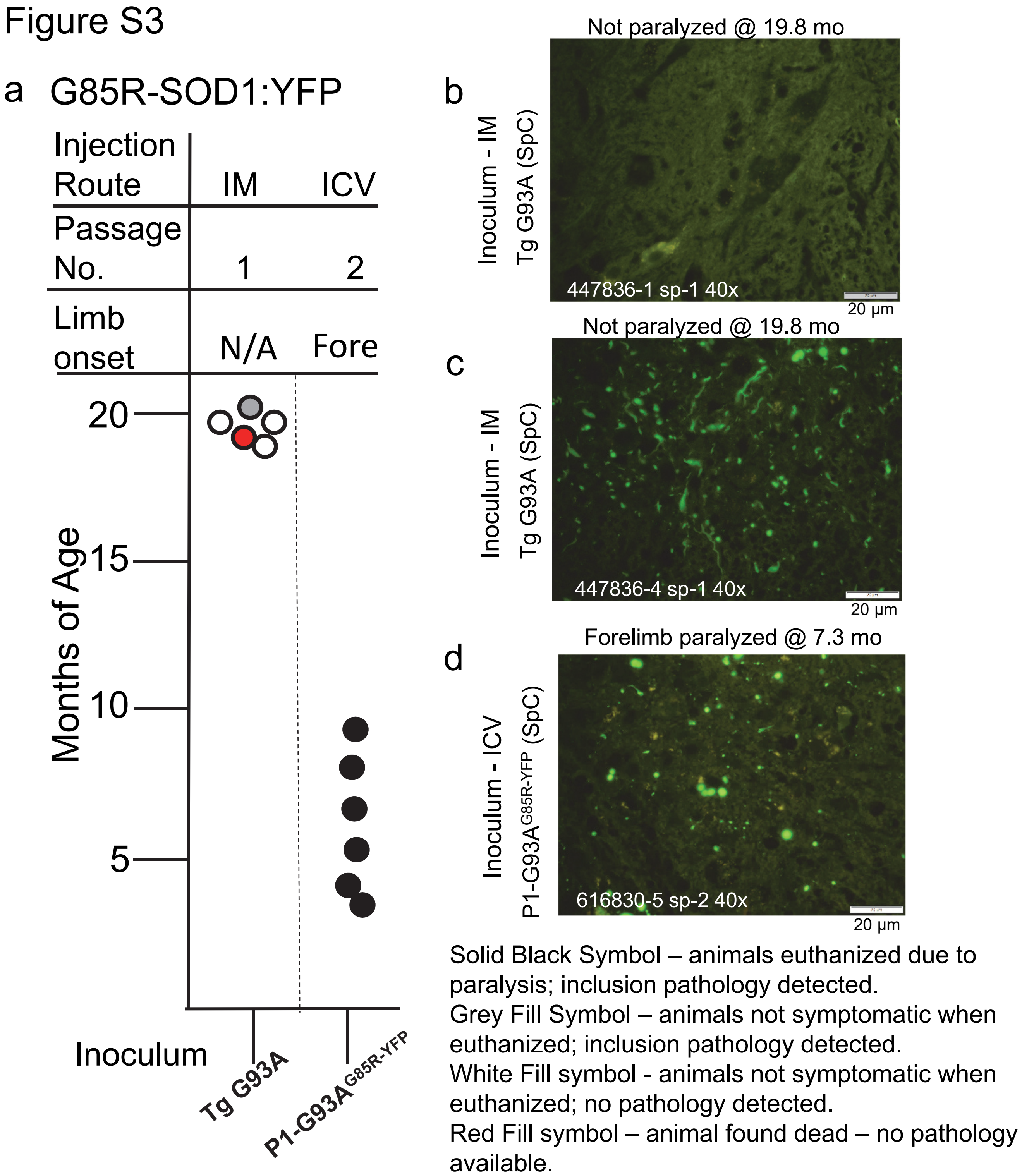

Fig. S3 Summary of seeding data for G85R-SOD1:YFP mice injected with homogenates from paralyzed mice by different routes (intramuscular vs intracerebral/intraventricular). a The age at which animals were euthanized either due to advanced age or paralysis is noted by each symbol. The legend for the symbols is provided in the figure. **b-d** Representative images of inclusion pathology in cryostat sections from mice seeded by the two different routes. The images shown are representative of the ventral horn of the lumbar or cervical spinal cord (2-3 sections per animal were visualized). The total number of animals examined and pathologically scored is documented in Supplemental Data File 1. Scale bars = 20 µm.

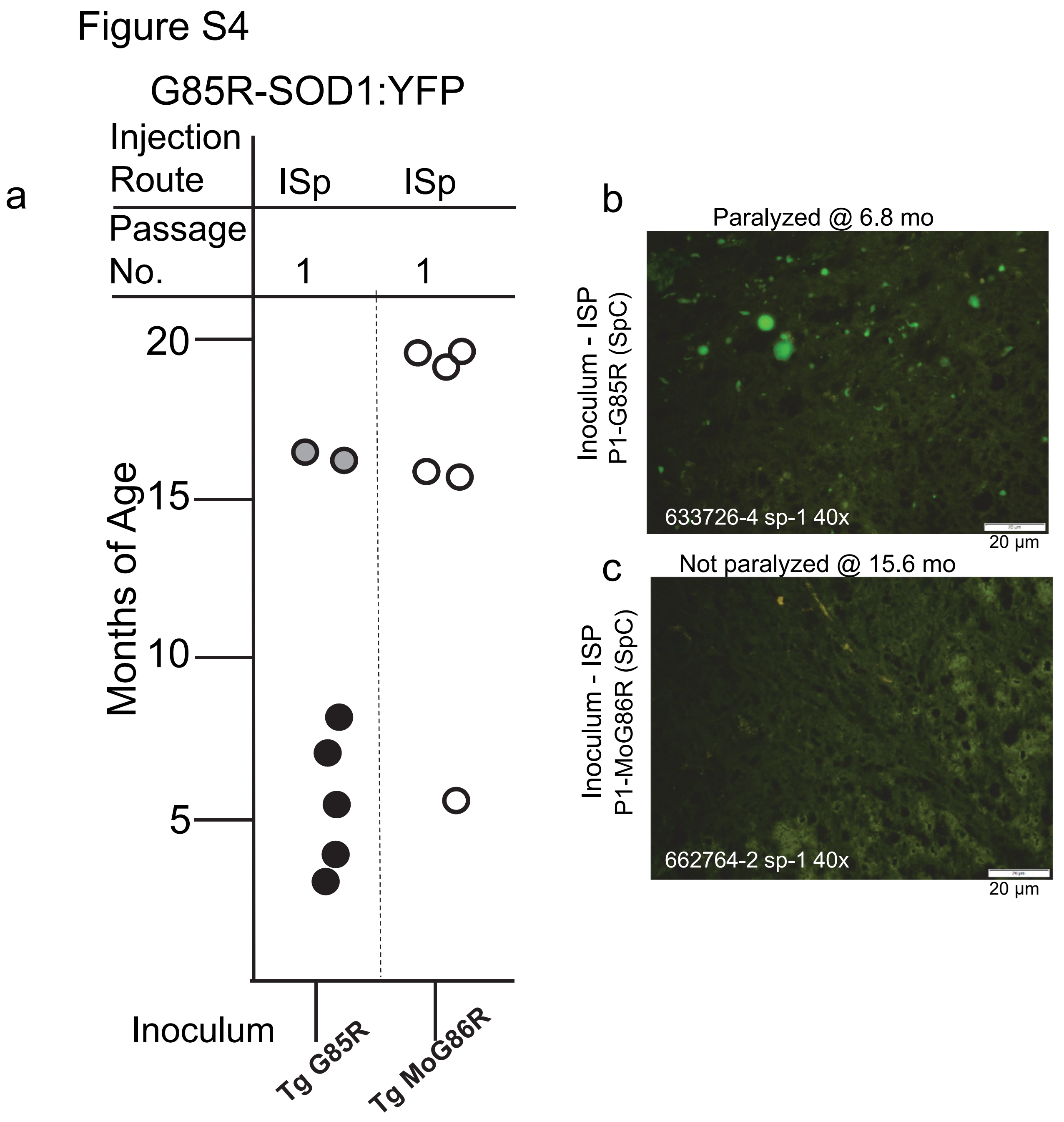

Fig. S4 Spinal homogenates from paralyzed murine G86R-SOD1 mice are efficient in seeding G85R-SOD1:YFP mice. a The age at which animals were euthanized either due to advanced age or paralysis is noted. Black circles indicate animals that developed paralsys. Gray circles indicate animals that were asymptomatic when euthanized but found to exhibit inclusion pathology. Open circles indicate mice that were asymptomatic at harvest and lacked any evidence of inclusion pathology. **b,c** Representative images of inclusion pathology seen in cryostat sections from mice that were paralyzed at the time of euthanasia or asymptomatic as noted. Mice injected with human G85R seeds produce punctate inclusions in the neuropil while mice injected with mouse G86R seeds lack inclusions. The images shown are representative of the ventral horn of the lumbar or cervical spinal cord (2-3 sections per animal were visualized). The total number of animals examined and pathologically scored is documented in Supplemental Data File 1. Scale bars = 20 µm.

Figure S5

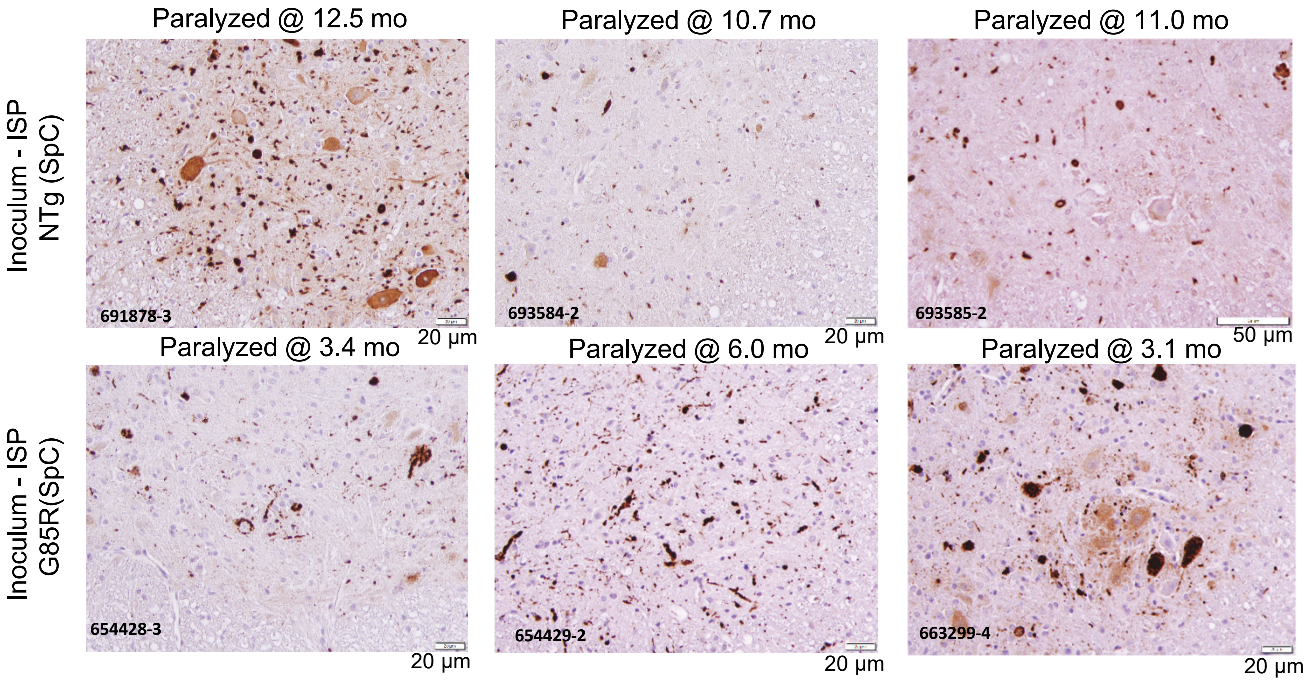

Fig. S5 Immunostaining of seeded untagged G85R-SOD1 mice with the C4F6 antibody to human G93A-SOD1. The images shown are representative of the ventral horn of the lumbar or cervical spinal cord in paraffin sections stained with C4F6 antibody (2-3 sections per animal were visualized). Mice that were injected with control NTg spinal cord homogenates are compared to mice injected with spinal homogenates from paralyzed G85R mice. The appearance of the pathologic inclusions is similar between the two groups, consisting of neuropil puncta with cell body accumulations. Scale bars = 20 or 50 µm as noted.

Supplemental Figure S6 (L126Z mice)

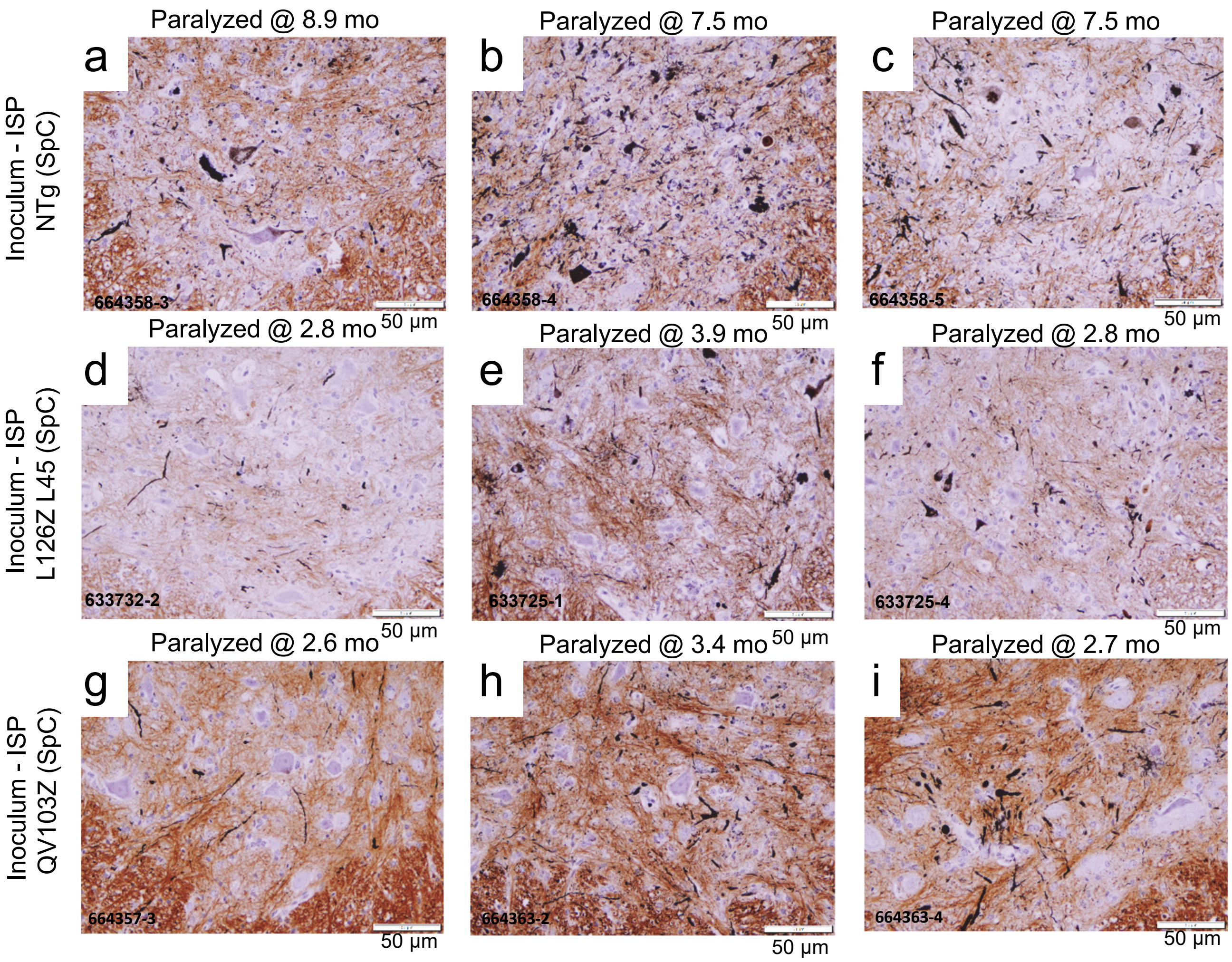

Fig. S6 Examples of inclusion pathology in seeded L126Z mice. The images shown are representative of the ventral horn of the lumbar or cervical spinal cord (2-3 sections per animal were visualized). Inclusion pathology was detected by CS-silver staining with H&E counterstaining. Inclusions appear as dark puncta in the neuropil with some cell body accumulation. The total number of animals examined and pathologically scored is documented in Supplemental Data File 1. Scale bars = 50 µm

Supplemental Figure S7 (QV103Z mice)

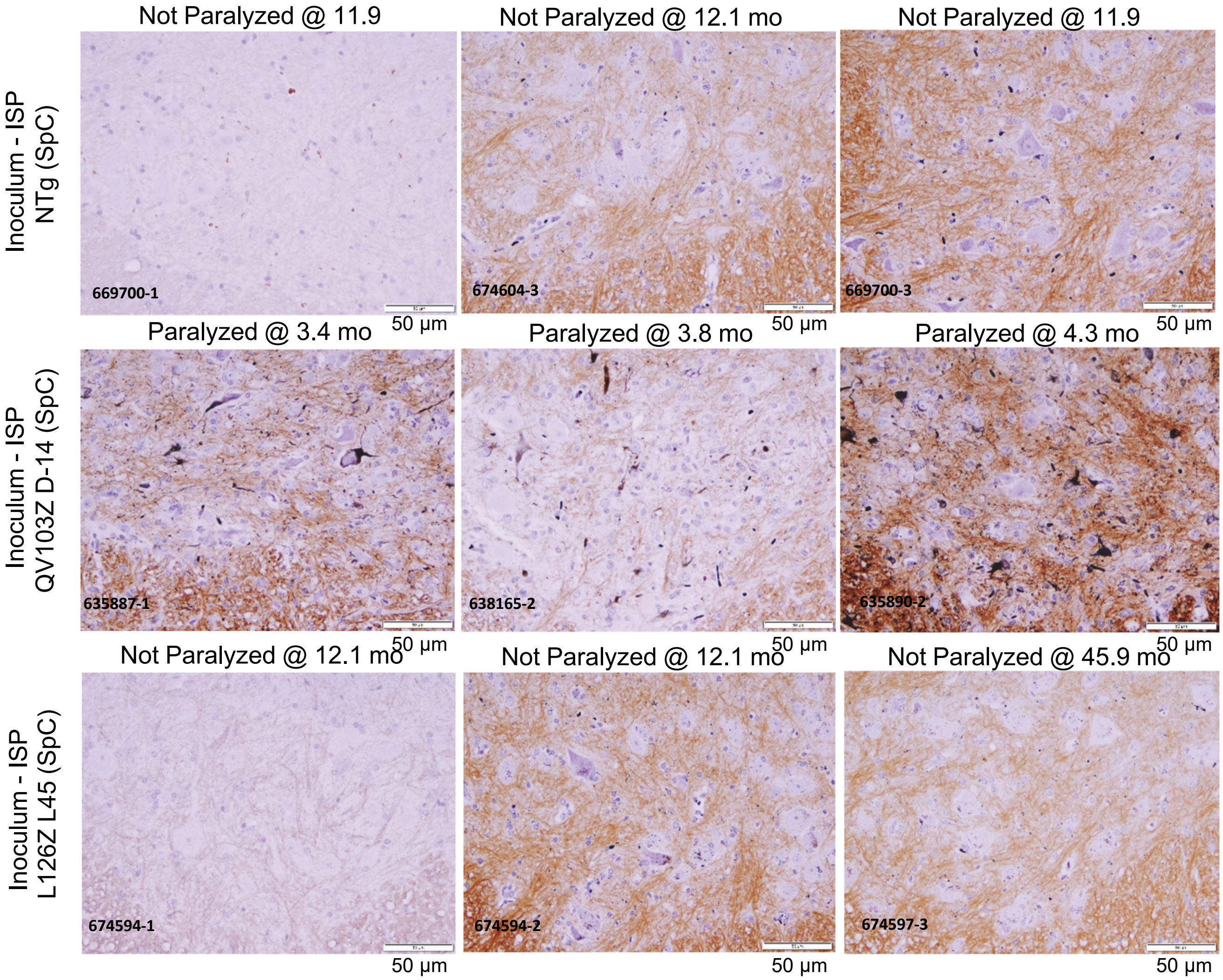

Fig. S7 Examples of inclusion pathology in seeded QV103Z mice. The images shown are representative of the ventral horn of the lumbar or cervical spinal cord (2-3 sections per animal were visualized). Inclusion pathology was detected by CS-silver staining with H&E counterstaining. Inclusions appear as dark puncta in the neuropil with some cell body accumulation. The total number of animals examined and pathologically scored is documented in Supplemental Data File 1.

Supplemental Figure S8 (Thy1-G93A) silver staining

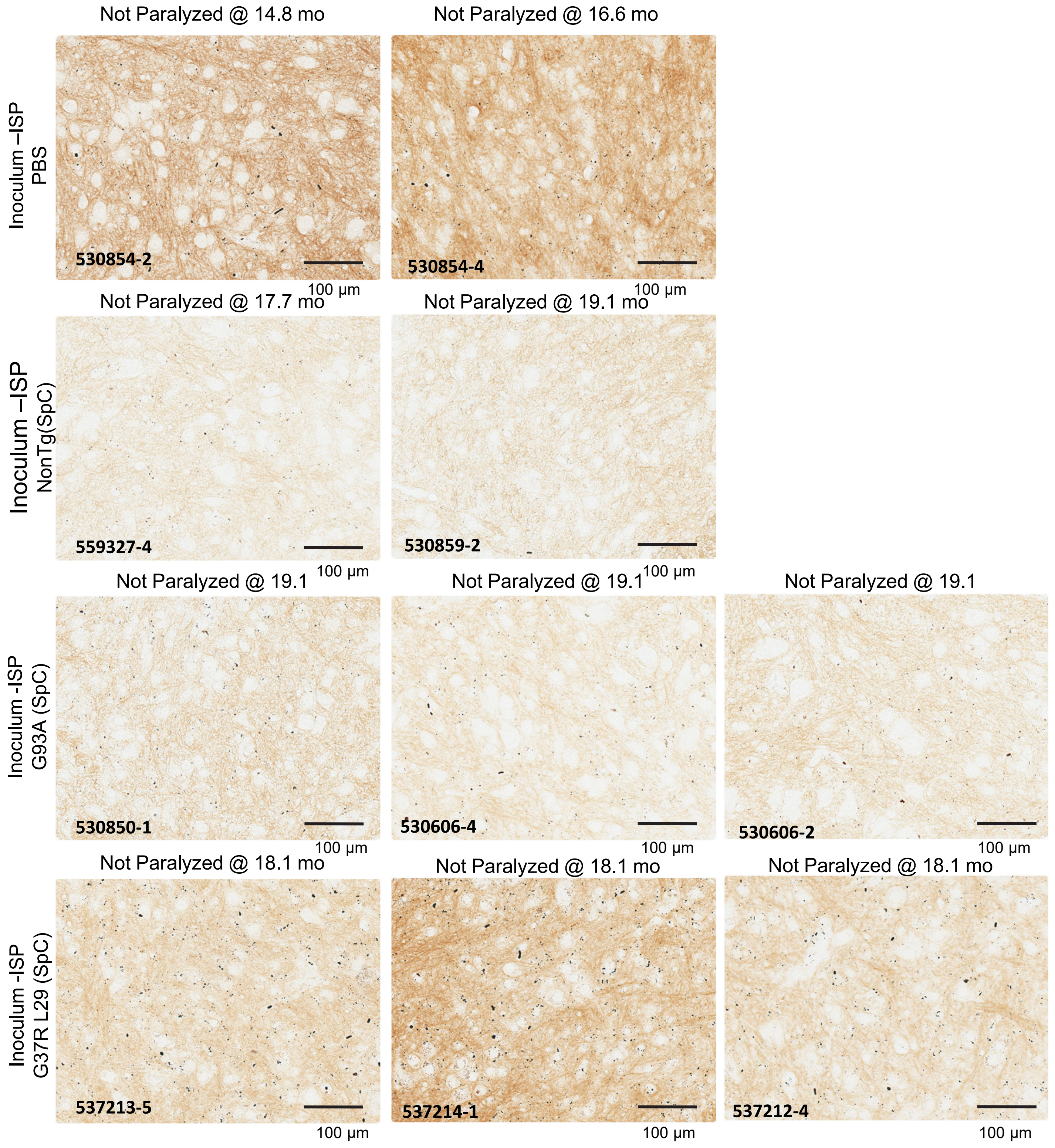

Fig. S8 Lack of inclusion pathology in asymptomatic Thy-1 G93A SOD1 mice seeded with spinal homogenates from paralyzed mutant SOD1 mice. The images shown are representative of the ventral horn of the lumbar or cervical spinal cord (2-3 sections per animal were visualized). Inclusion pathology was detected by CS-silver staining. These older animals have variable levels of discrete argentophilic puncta, but lack clearly definable inclusions. The total number of animals examined and pathologically scored is documented in Supplemental Data File 1. Scale bars = 50 µm.

Supplemental Figure S9 (VLE-G93A)

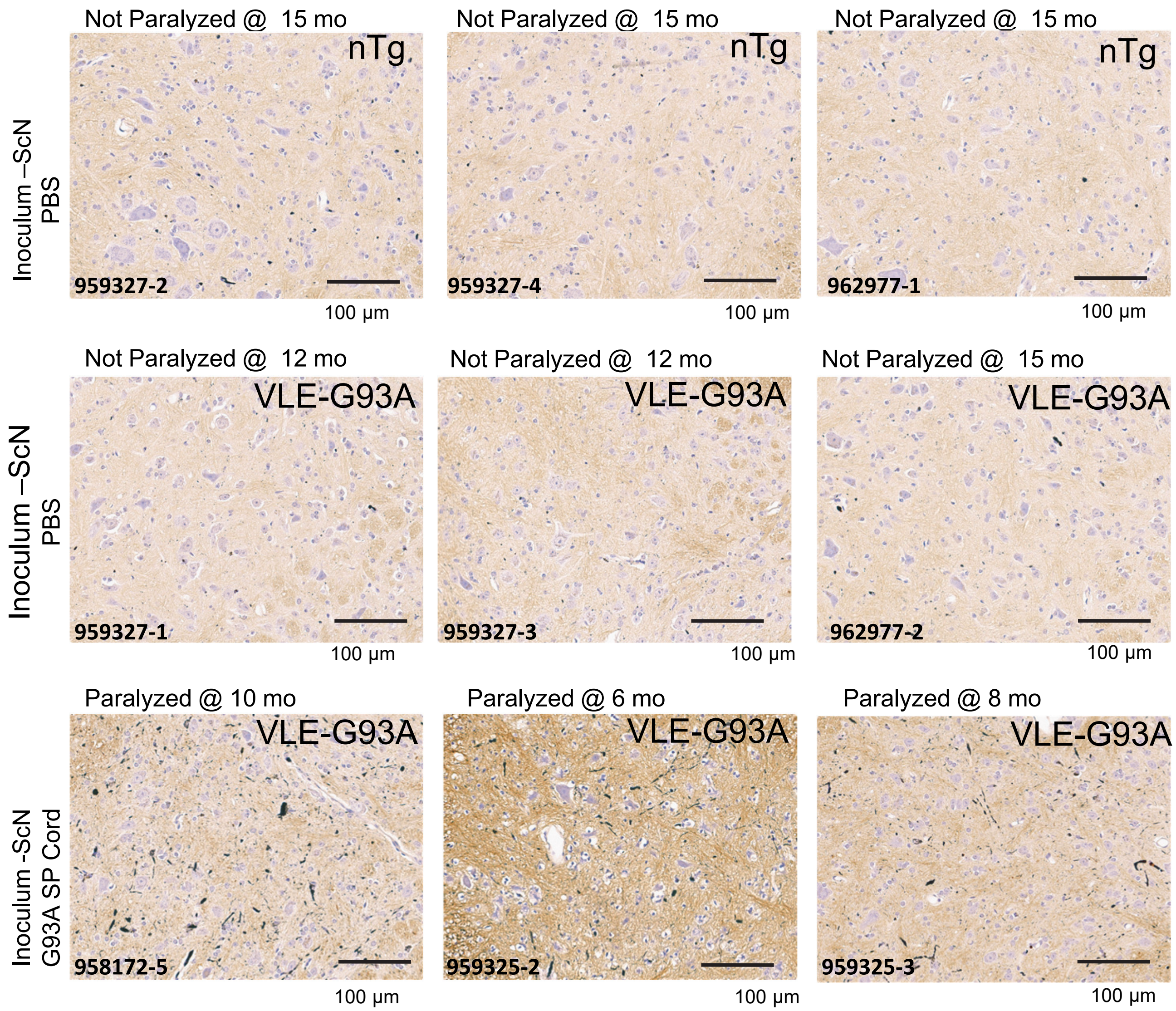

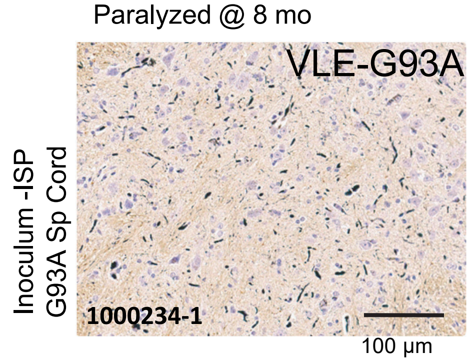

Fig. S9 Examples of inclusion pathology in asymptomatic and paralyzed VLE-G93A SOD1 mice seeded with spinal homogenates from paralyzed mutant SOD1 mice. The images shown are representative of the ventral horn of the lumbar or cervical spinal cord (2-3 sections per animal were visualized). Inclusion pathology was detected by CS-silver staining with H&E counterstaining. In paralyzed VLE-G93A mice, inclusions appear as dark puncta in the neuropil with some cell body accumulation. The total number of animals examined and pathologically scored is documented in Supplemental Data File 1. Scale bars = 100 µm.

Supplemental Figure S10 PrP.G37R-110

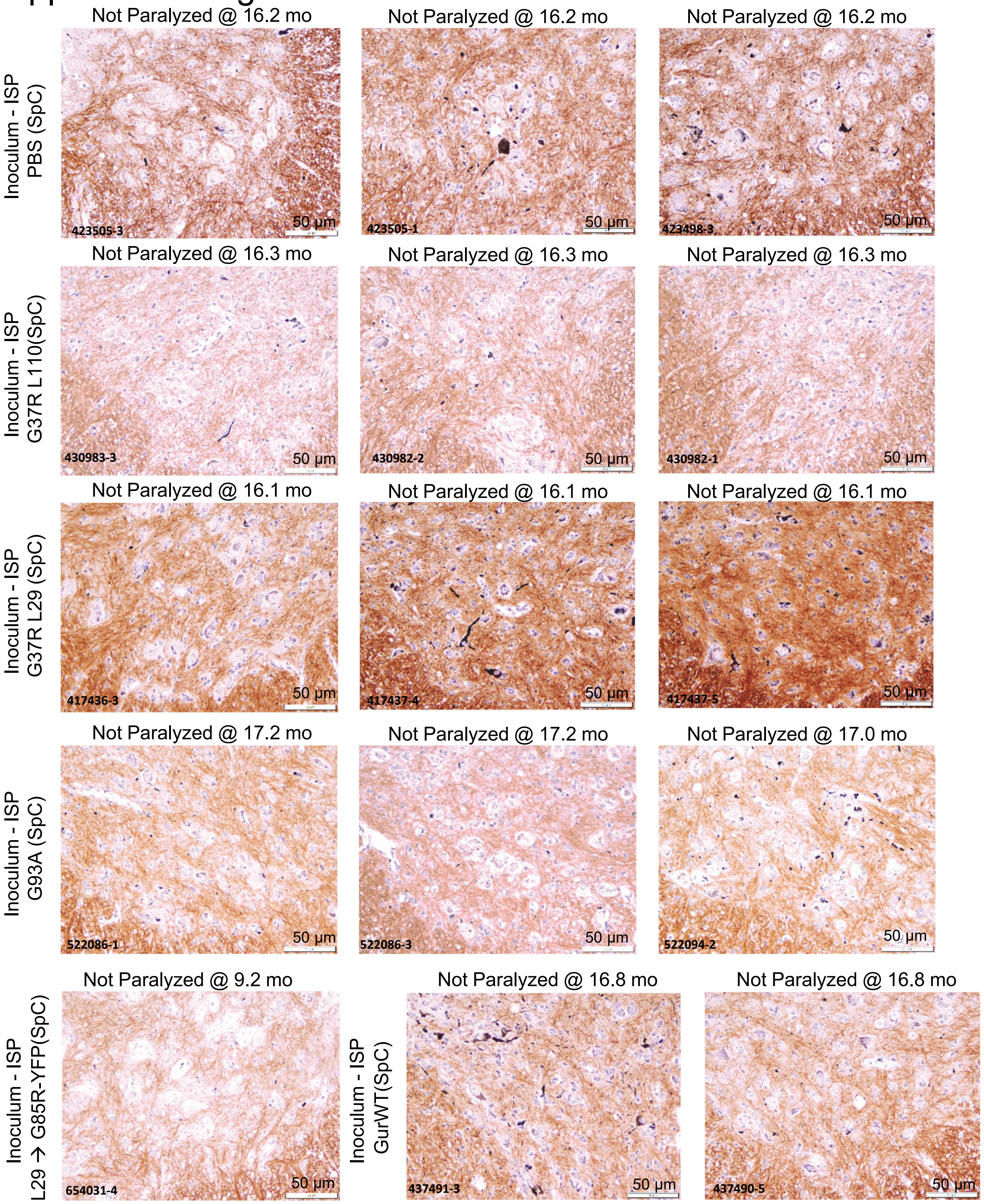

Fig. S10 Lack of inclusion pathology in asymptomatic PrP-G37R SOD1 mice seeded with spinal homogenates from paralyzed mutant SOD1 mice. The images shown are representative of the ventral horn of the lumbar or cervical spinal cord (2-3 sections per animal were visualized). Inclusion pathology was detected by CS-silver staining with H&E counterstaining. These older PrP-G37R animals have variable levels of punctate neuropil inclusions. The frequency of such inclusions was not obviously elevated in seeded mice as compared to mice injected with PBS. The total number of animals examined and pathologically scored is documented in Supplemental Data File 1. Scale bars = 50 μm.

Supplemental Figure S11

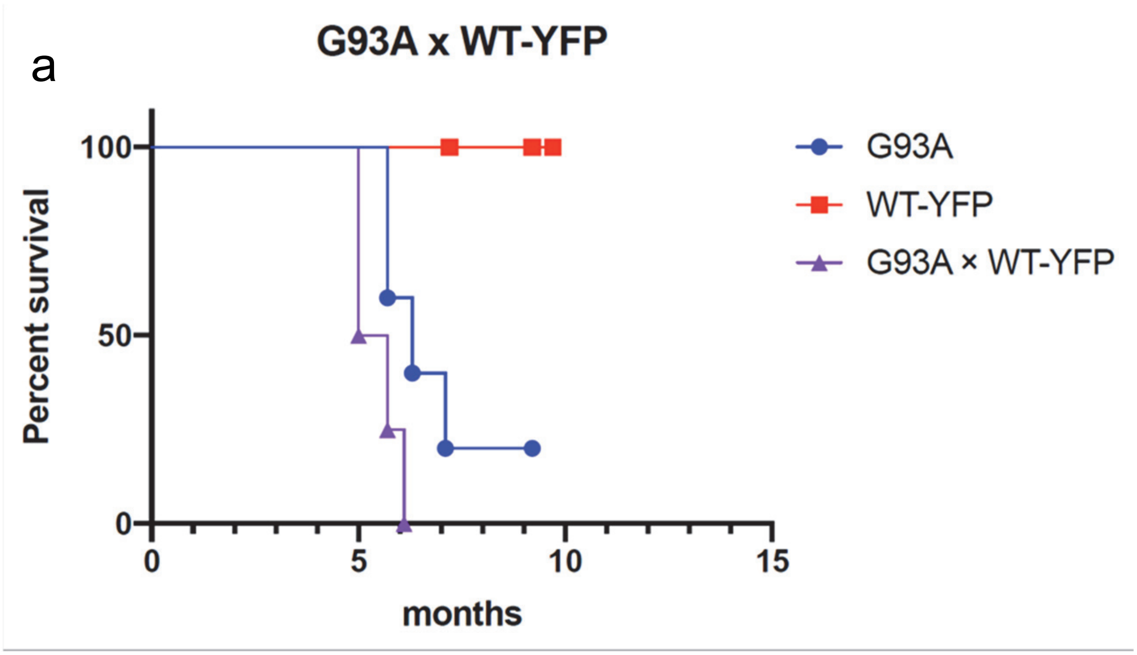

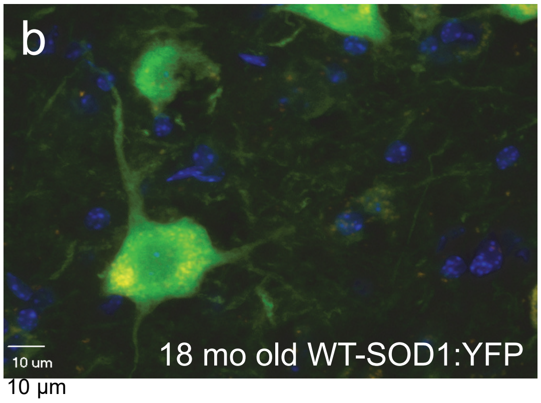

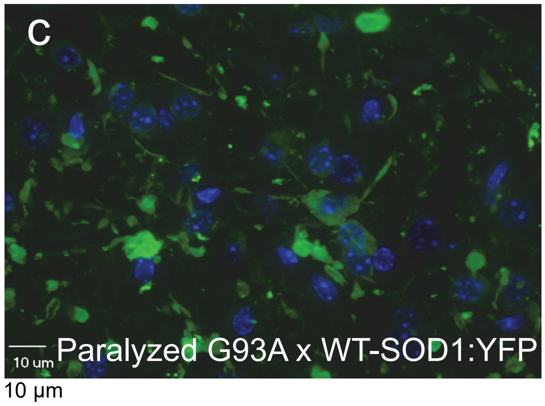

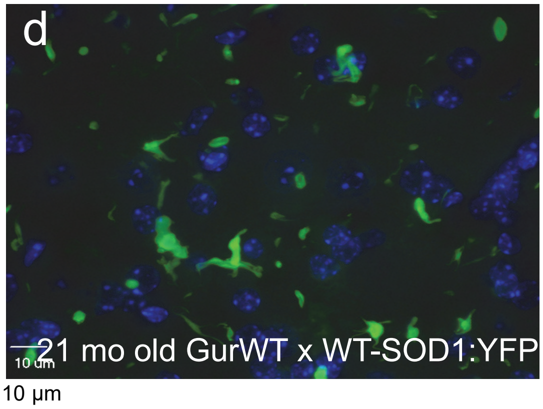

Fig. S11 Induction of WT-SOD1:YFP inclusion pathology by the co-expression of mutant or WT untagged human SOD1. a Kaplan-Meier plot of survival (age to bilateral hindlimb paralysis) for G93A mice crossed to WT-SOD1:YFP mice. **b,c** Representative pathologic images from aged WT-SOD1:YFP mice (n = 8) and paralyzed bigenic WT-SOD1:YFP mice co-expressing G93A SOD1 (n = 4). **d** Representative image of inclusion pathology in mice co-expressing WT human SOD1 (GurWT mice) and WT-SOD1:YFP (n = 8). Images are representative of 2-3 sections per mouse. Scale bars = 10 μm.

Supplemental Figure S12

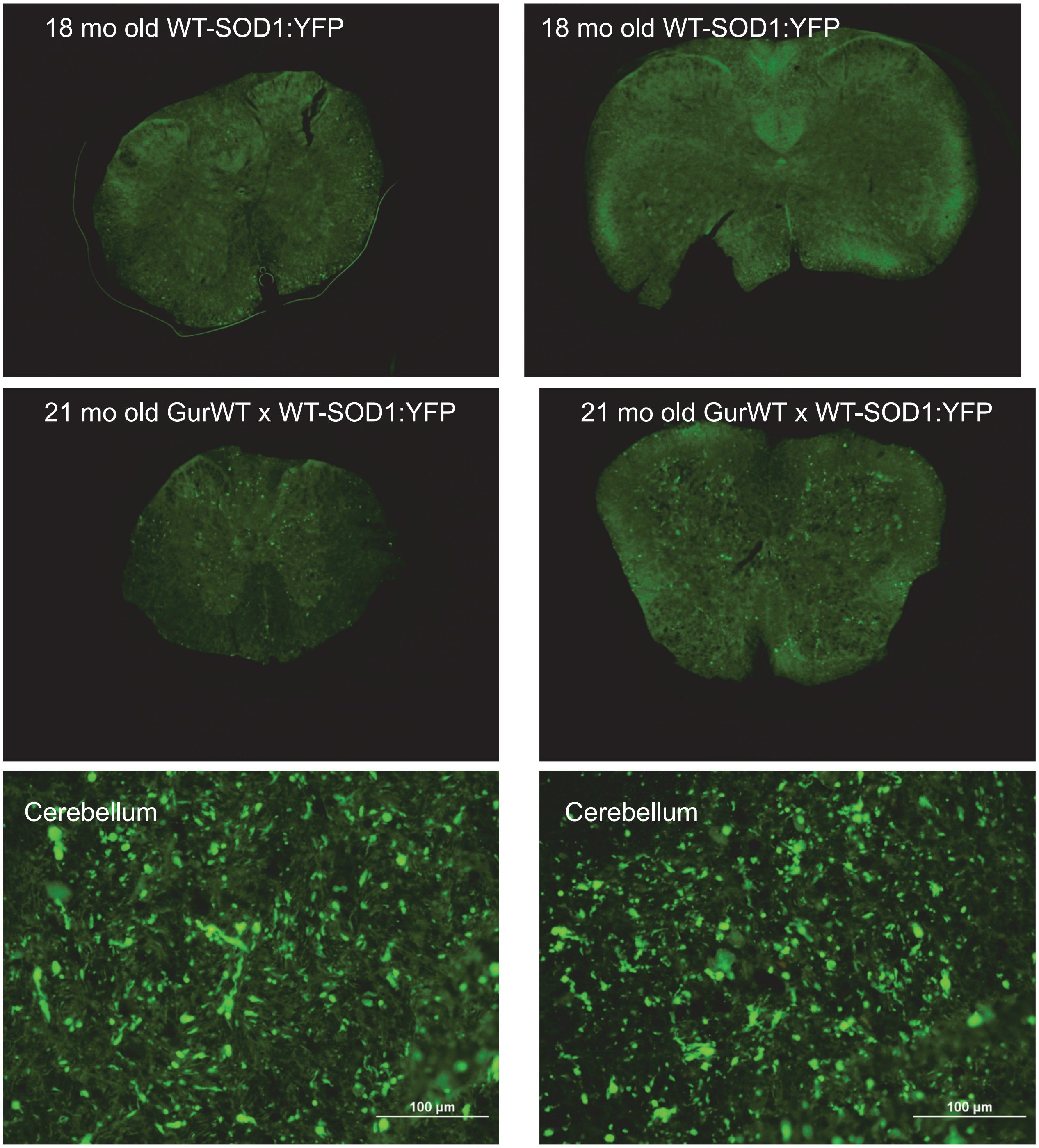

Fig. S12 Cerebellar inclusion pathology in bigenic mice that co-express untagged WT human SOD1 with WT-SOD1:YFP. The images shown are low power views of the entire spinal cords from 18-month-old WT-SOD1:YFP mice or bigenic GurWT/WT-SOD1:YFP mice. Punctate inclusion pathology can be seen in the spinal cord of the bigenic mice. Higher power views of the dentate of the cerebellum from bigenic GurWT/WT-SOD1:YFP mice are shown in the bottom panels. The total number of animals examined and pathologically scored is the same as described in Figure 9. Scale bars = 100 µm.

Supplemental Figure S13 WT-SOD1:YFP

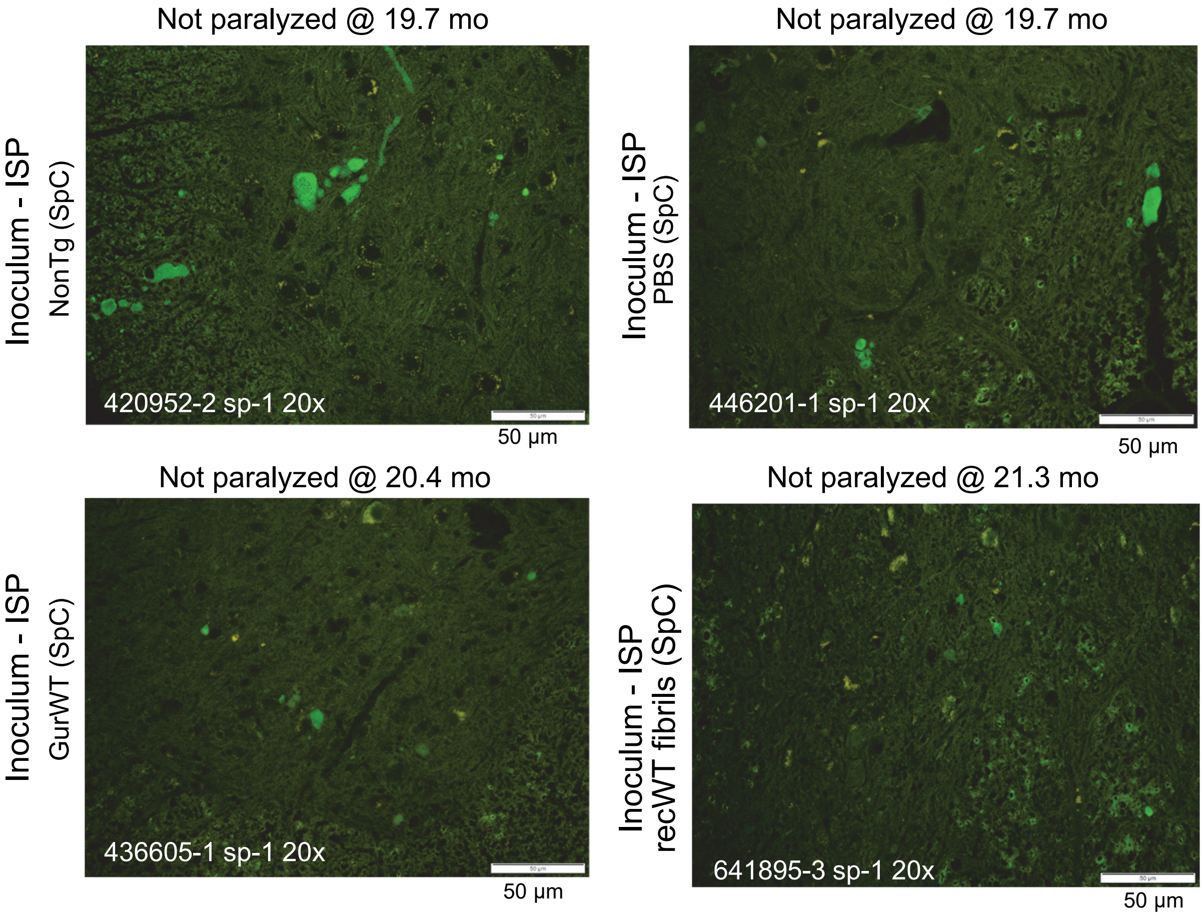

Fig. S13 Analysis of inclusion pathology in asymptomatic WT-SOD1:YFP mice seeded with spinal homogenates from aged GurWT mice. YFP fluorescence images were captured as described in Methods. The images shown are representative of the ventral horn of the lumbar or cervical spinal cord (2-3 sections per animal were visualized). The total number of animals examined and pathologically scored is documented in Supplemental Data File 1. Scale bars = 50 µm.

Supplemental Figure S14 GurWT

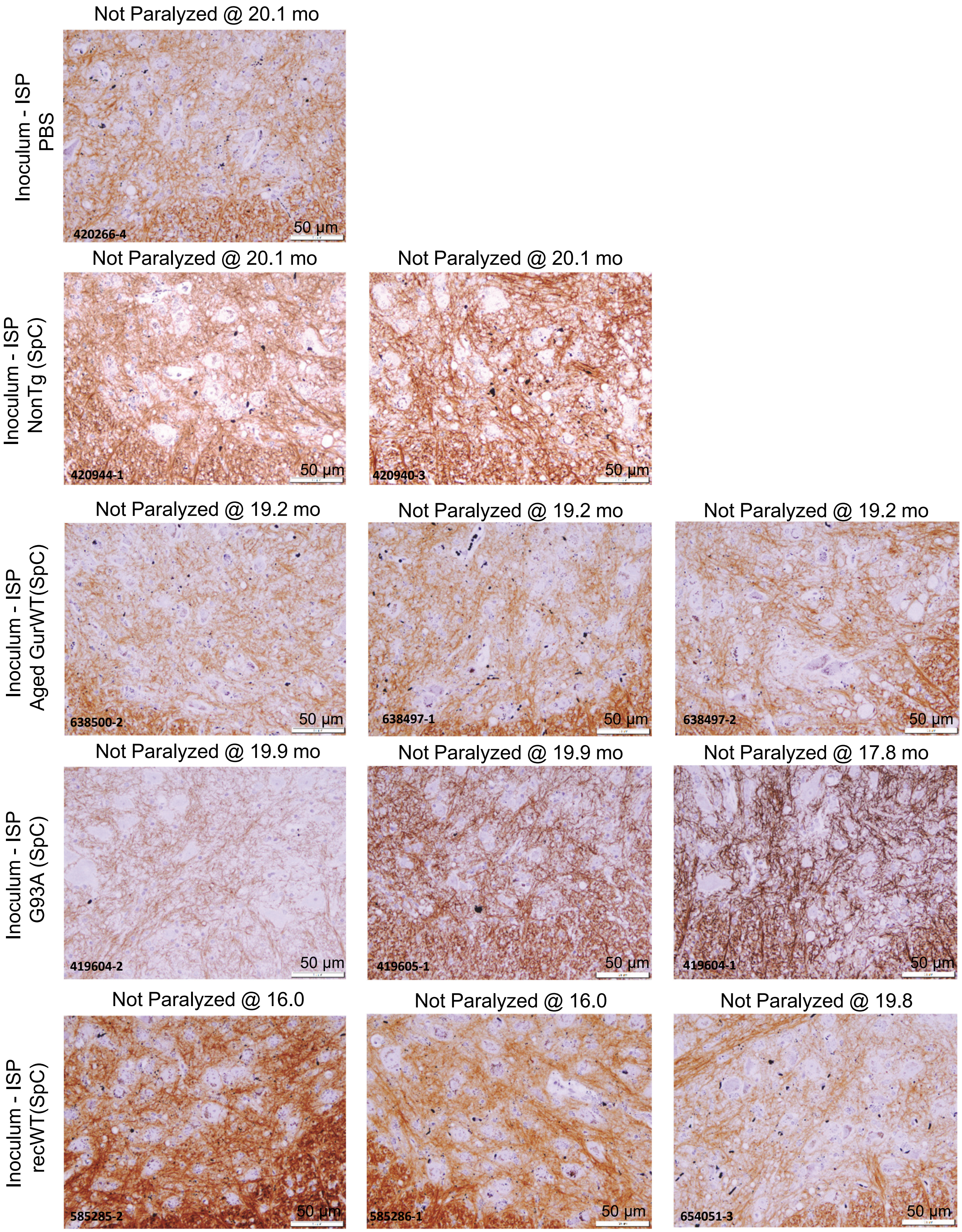

Fig. S14 Lack of inclusion pathology in asymptomatic GurWT SOD1 mice seeded with spinal homogenates from paralyzed mutant SOD1 mice. The images shown are representative of the ventral horn of the lumbar or cervical spinal cord (2-3 sections per animal were visualized). Inclusion pathology was detected by CS-silver staining with H&E counterstaining. These older animals have variable levels of discrete argentophilic puncta, but lack clearly definable inclusions. The total number of animals examined and pathologically scored is documented in Supplemental Data File 1. Scale bars = 50 μm.

Figure S15

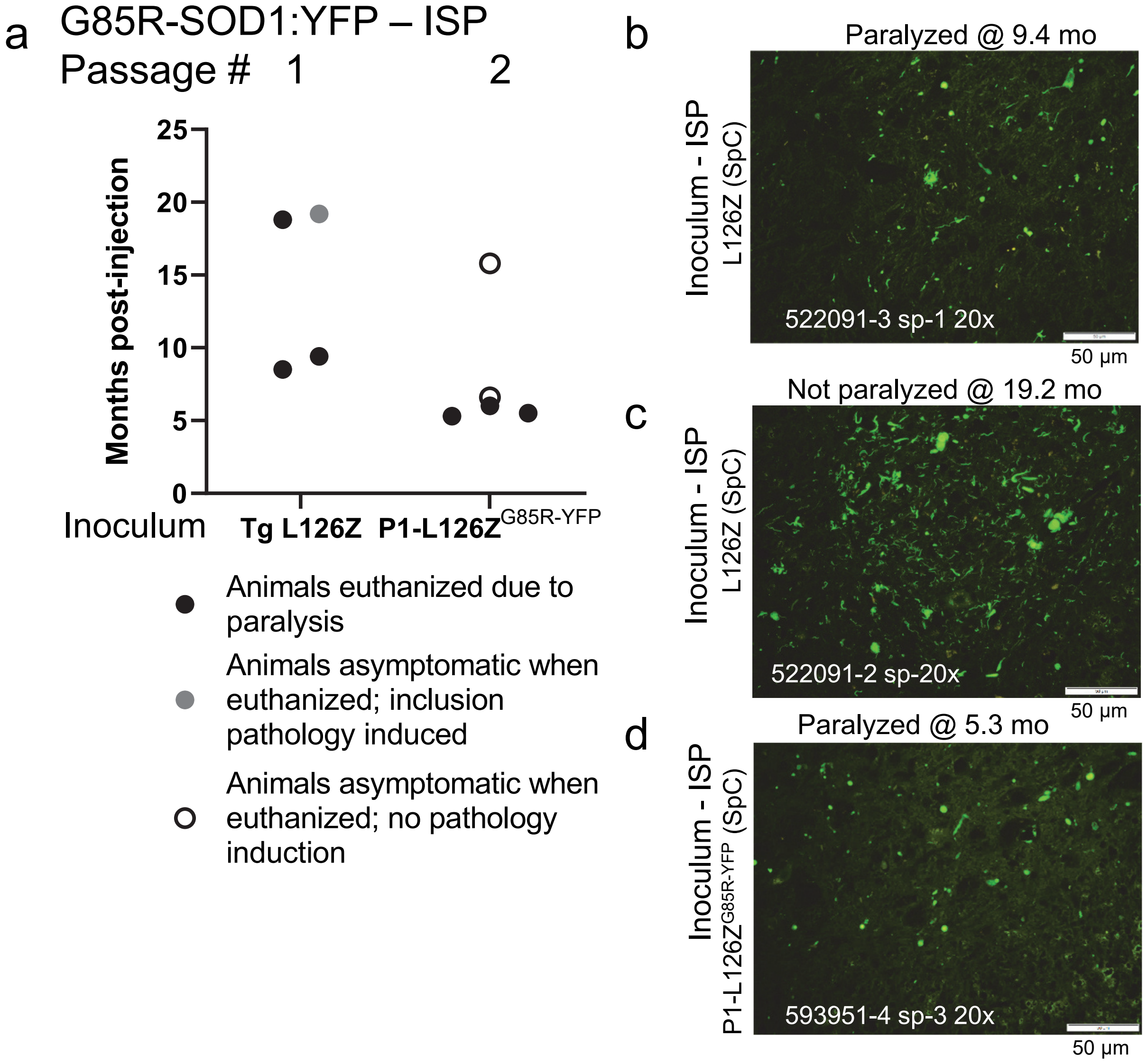

Fig. S15 Summary of previous study of G85R-SOD1:YFP mice seeded by spinal homogenates from paralyzed L126Z mice. a The age at which animals were euthanized either due to advanced age or paralysis is noted by they symbols. The legend for the symbols is provided in the figure. **b-d** Representative images of inclusion pathology in mice that were paralyzed or asymptomatic at the time of euthanasia as noted. Mice injected with human L126Z seeds produce a mixture of punctate and fiber-like inclusions in the neuropil. Second passage of the seeds was moderately efficient in a small cohort of animals. The images shown are representative of the ventral horn of the lumbar or cervical spinal cord from paraffin sections (2-3 sections per animal were visualized). The total number of animals examined and pathologically scored is documented in Supplemental Data File 1. Scale bars = 50 µm. Data previously published in a different form in [17].
